# Duplicated *dnmt3aa* and *dnmt3ab* DNA Methyltransferase Genes Play Essential and Non-Overlapped Functions on Modulating Behavioral Control in Zebrafish

**DOI:** 10.3390/genes11111322

**Published:** 2020-11-07

**Authors:** Yu-Heng Lai, Gilbert Audira, Sung-Tzu Liang, Petrus Siregar, Michael Edbert Suryanto, Huan-Chau Lin, Omar Villalobos, Oliver B. Villaflores, Erwei Hao, Ken-Hong Lim, Chung-Der Hsiao

**Affiliations:** 1 Department of Chemistry, Chinese Culture University, Taipei 11114, Taiwan; lyh21@ulive.pccu.edu.tw; 2 Department of Chemistry, Chung Yuan Christian University, Chung-Li 320314, Taiwan; gilbertaudira@yahoo.com (G.A.); siregar.petrus27@gmail.com (P.S.); 3 Department of Bioscience Technology, Chung Yuan Christian University, Chung-Li 320314, Taiwan; stliang3@gmail.com (S.-T.L.); michael.edbert93@gmail.com (M.E.S.); 4 Division of Hematology and Oncology, Department of Internal Medicine, Mackay Memorial Hospital, Number 92, Section 2, Chungshan North Road, Taipei 10449, Taiwan; hcanduhmmh@gmail.com; 5 Department of Pharmacy, Faculty of Pharmacy, University of Santo Tomas, Manila 1015, Philippines; oavillalobos@ust.edu.ph; 6 Department of Biochemistry, Faculty of Pharmacy, University of Santo Tomas, Manila 1015, Philippines; obvillaflores@ust.edu.ph; 7 Guangxi Scientific Experimental Center of Traditional Chinese Medicine, Guangxi University of Chinese Medicine, Nanning 530200, Guangxi, China; 8 Guangxi Key Laboratory of Efficacy Study on Chinese Materia Medica, Guangxi University of Chinese Medicine, Nanning 530200, Guangxi, China; 9 Department of Medicine, MacKay Medical College, Sanzhi Dist., New Taipei City 252, Taiwan; 10 Center of Nanotechnology, Chung Yuan Christian University, Chung-Li 320314, Taiwan

**Keywords:** *Danio rerio*, anxiety, behavior, DNA methylation profiling, epigenetic

## Abstract

DNA methylation plays several roles in regulating neuronal proliferation, differentiation, and physiological functions. The major de novo methyltransferase, DNMT3, controls the DNA methylation pattern in neurons according to environmental stimulations and behavioral regulations. Previous studies demonstrated that knockout of *Dnmt3* induced mouse anxiety; however, controversial results showed that activation of *Dnmt3* causes anxiolytic behavior. Thus, an alternative animal model to clarify *Dnmt3* on modulating behavior is crucial. Therefore, we aimed to establish a zebrafish (*Danio rerio*) model to clarify the function of *dnmt3* on fish behavior by behavioral endpoint analyses. We evaluated the behaviors of the wild type, *dnmt3aa*, and *dnmt3ab* knockout (KO) fish by the novel tank, mirror biting, predator avoidance, social interaction, shoaling, circadian rhythm locomotor activity, color preference, and short-term memory tests. The results indicated that the *dnmt3aa* KO fish possessed abnormal exploratory behaviors and less fear response to the predator. On the other hand, *dnmt3ab* KO fish displayed less aggression, fear response to the predator, and interests to interact with their conspecifics, loosen shoaling formation, and dysregulated color preference index ranking. Furthermore, both knockout fishes showed higher locomotion activity during the night cycle, which is a sign of anxiety. However, changes in some neurotransmitter levels were observed in the mutant fishes. Lastly, whole-genome DNA methylation sequencing demonstrates a potential network of Dnmt3a proteins that is responsive to behavioral alterations. To sum up, the results suggested that the *dnmt3aa* KO or *dnmt3ab* KO fish display anxiety symptoms, which supported the idea that Dnmt3 modulates the function involved in emotional control, social interaction, and cognition.

## 1. Introduction

DNA methylation plays an important role in epigenetic modification [1]. The add on and off of methyl group onto DNA without altering protein-coding sequences is catalyzed by DNA methyltransferases (DNMTs) and demethylase (TETs) [2,3]. The family of *Dnmt* homolog genes, *Dnmt1*, *Dnmt2*, *Dnmt3A*, *Dnmt3B*, and *Dnmt3L*, have been identified in mammals [4,5]. In general, *Dnmt1* plays a role in DNA methylation maintenance, while *Dnmt3a, Dnmt3b*, and *Dnmt3L* take charge of performing de novo methylation [6,7,8]. *Dnmt2* is known for its catalytic role on cysteine methylation; however, the precise function is still ambiguous [9]. Due to the importance of DNMTs in genomic modification, dysregulation on DNMTs may result in inherited diseases, such as chromosome instability [10], developmental abnormality [11], and cancer formation [12,13].

Previous evidence has shown that neuronal diseases, such as Alzheimer’s disease, anxiety, major depression, and Schizophrenia are closely related to dysregulation of DNA methylation [14,15,16,17] or *Dnmt3* expression level in the brain [12,16]. Previous studies showed that changes in DNA methylation in brain tissue affect learning and memorial behaviors in the mouse models [18,19]. The *Dnmt3a* plays an important role in de novo DNA methylation in mice, which is modulated by environmental factors to regulate downstream genes and affects behaviors [20]. Moreover, the epigenetic process was implicated to associate with long-term memory [21,22]. For example, mice with forebrain-specific *Dnmt3a* deficiency displayed deficits in long-term potentiation leading to behavioral disorders [23]. However, *Dnmt1* knockout (KO) mice displayed an anxiolytic and anti-depression phenotype, while *Dnmt3a* deficiency does not alter behavior, which indicated *Dnmt1* and *Dnmt3a* played distinct roles in controlling emotion in mice [23,24]. On the contrary, *Dnmt3a* deficiency within the prefrontal cortex showed an increase of anxiety-like actions [12], which indicated *Dnmt3a* function remained controversial. Therefore, considering the complexity of the mouse model, establishing an alternative animal model to clarify *Dnmt3* on modulating behavior by a series of endpoint analyses is crucial.

Zebrafish (*Danio rerio*), a lower vertebrate model for behavioral study with great potential in the latest years, was popular in studying behavioral genetics since its whole genome has been decoded. The genomic pattern and chromosomal localization of five DNMT-related genes in zebrafish have been successfully identified [24]. Zebrafish encodes multiple homologs of mammalian *Dnmt3a* (*dnmt3a1* and *dnmt3a2*) and *Dnmt3b* (*dnmt3b1*, *3b2*, *3b3*, and *3b4*) genes that occur in the teleost fish lineage shortly after their divergence from the tetrapod lineage. Duplicated genes in the zebrafish model provide a unique opportunity for obtaining new mechanistic insights into the multiple functions of a gene family [25]. In addition, functional genomic tools to perform gain-of-function (i.e., Tol2 transposon) and loss-of-function (i.e., TALEN and CRISPR genome editing tools) approaches have been robustly developed and applied in zebrafish models. Zebrafish demonstrate robust behavioral responses and evolutionarily conservation to mammalian species [26]. Nowadays, with the computational tools for locomotion tracking, various zebrafish behavior endpoints are easier ] quantify. Thus, combined with TALEN techniques used for gene knockout of either *dnmt3aa* or *dnmt3ab* gene, this study aimed to explore the behavior-related functions of two human *Dnmt3a* homolog genes in zebrafish by conducting multiple behavior tests and performing DNA methylation profiling. By applying the systematic investigation of behaviors and genomic analysis, hopefully, the *Dnmt*-associated network will be elucidated and may apply to neurogenetic research in the near future.

## 2. Materials and Methods

### 2.1. Animal Ethics

All the experimental protocols and procedures involving zebrafish were approved by the Committee for Animal Experimentation of the Chung Yuan Christian University (CYCU) (Number: CYCU104024, issue date 21 December 2015). All experiments were performed in accordance with the guidelines for laboratory animals issued by the Institutional Animal Care and Use Committees (IACUCs) of CYCU.

### 2.2. Fish Lines and Husbandry

**Figure 1:**
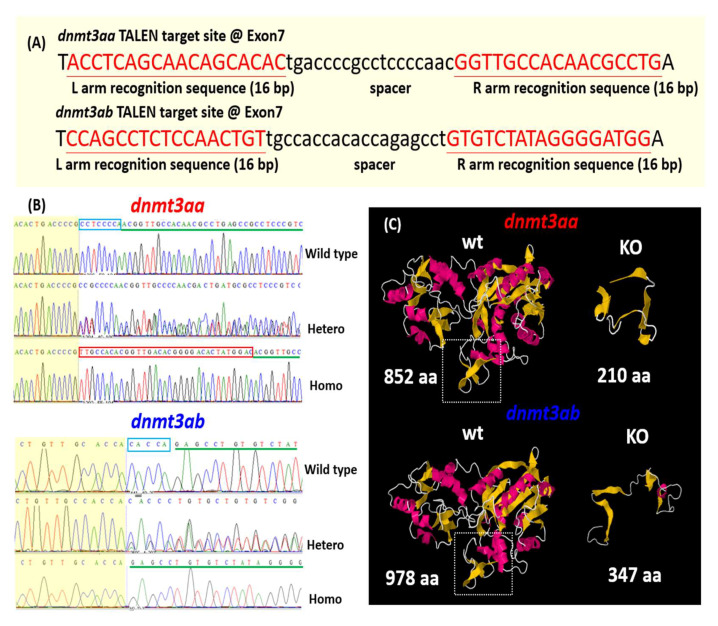
Generation of *dnmt3aa* and *dnmt3ab* gene knockout (KO) zebrafish by using TALEN (transcription activator-like effector nuclease) genome-editing tool. (**A**) The location of TALEN right arm, spacer, and left arm on targeting zebrafish *dnmt3aa* and *dnmt3ab* genes are showed. (**B**) The Sanger sequencing dendrogram showed the sequence of wildtype, heterozygotes, and homozygotes of *dnmt3aa* (upper panel) and *dnmt3ab* (lower panel) mutants. (**C**) Three-dimension model predictions on the structure of wild type and Dnmt3aa and Dnmt3ab mutated proteins.

TALEN technique was used to create zebrafish mutants that defected in *dnmt3aa* (ENSDARG00000005394) and *dnmt3ab* (ENSDARG00000015566) genes. The custom-design TALEN vectors were purchased from Zgenebio Inc. (Taipei, Taiwan) and the TALEN target site was designed to target exon 7 and exon 12 for zebrafish *dnmt3aa* and *dnmt3ab* genes, respectively (Figure 1A). The TALEN target sequences were 5′-ACCTCAGCAACAGCACACtgaccccgcctccccaacGGTTGCCACAACGCCTG-3′ for *dnmt3aa* and 5′-CCAGCCTCTCCAACTGTtgccaccacaccagagcctGTGTCTATAGGGGATGG-3′ for *dnmt3ab* genes (TALEN recognition sites are capitalized and underlined while sequence between two recognition sites is the spacer). After injection with the 5′-capping in vitro synthesized TALEN left and right arm mRNAs, the injected embryos were raised to adulthood and mated with wild-type fish to generate F1 progeny. Afterward, Sanger sequencing was performed to screen the potential mutant carriers and cross the F1 pairs with the same genotype to generate homozygotic F2 progenies. Finally, we identified *dnmt3aa* mutant carrying 8bp deletion and 25 bp insertion (5′-CCTCCCCA-3′ deletion and 5′-TTGCCACACGGTTGACACGGGGAAACTATGGAC-3′ insertion) and *dnmt3ab* mutant carrying 5 bp deletion (5′-CACCA-3′ deletion) (Figure 1B). The corresponding translated protein sizes for mutated *dnmt3aa* and *dnmt3ab* genes were shortened from 852 aa to 210 aa and from 978 aa to 347 aa, respectively. The predicted 3D protein structure was analyzed by using an online tool of PHYRE2 (http://www.sbg.bio.ic.ac.uk/phyre2/html/page.cgi?id=index) and mutated *dnmt3aa* and *dnmt3ab* encoded two internal repeated domains at N-terminus. The key functional domains within Dnmt3a such as PWWP, RING, and DNA methylase domains were all missing (Figure 1C).

AB strain zebrafish were held and raised in a trapezoid tank with 34 cm at the top, 23 cm along the bottom, 19 cm along the diagonal side, 18 cm high, and 27 cm wide filled with 8 L of filtered water. The condition was maintained at 25 ± 1 °C with a 14/10 h light/dark cycles in culture water (UV sterilized and well-aerated water, pH 7.2 + 0.4, dissolved oxygen, 6.5 ± 0.2 mg/L, electrical conductivity, 0.254 ± 0.004 mS/cm, water hardness, 183 ± 5 mg of CaCO_3_/L). Fish were fed twice a day with either commercial dry food or brine shrimps. Maintenance and routine culture were based on the previously described method [27]. Adult zebrafish of both sexes (≈6 months) with a healthy condition were used in the current study. Two generations of each fish line were used in this study. The first generation was used in several behavior tests, which were novel tank, mirror biting, predator avoidance, social interaction, and shoaling tests. Meanwhile, their offspring, which were the second generation, were used for other assays, including morphometric, circadian rhythm locomotor activity, color preference, short-term memory, and biochemical assays. The whole test was divided into two parts to maintain the consistency of zebrafish age during the test. While the amount of successfully injected *dnmt3ab* KO fish in the first generation was numerous (*n* > 30), slightly fewer *dnmt3aa* KO fish were obtained in the first generation (*n* = ~10). However, based on several prior studies, this sample size was sufficient to conduct various behavioral tests properly [28,29,30,31,32]. Nevertheless, a higher *n* number of fish was generated in the second generation.

### 2.3. Morphometric Analysis

The images of WT and KO fish were captured, and the image files were converted to .tps file type by using TpsUtil for morphometric analysis (http://life.bio.sunysb.edu/morph/soft-utility.html). Later, the image landmark was digitized by the TpsDig2 toll and procrustes analysis for zebrafish was performed by using MorphoJ software which can generate a covariance matrix (http://www.flywings.org.uk/morphoj_page.htm). Afterward, principal component analysis (PCA) was performed to compare the morphometric difference between the WT and mutant fish as described in the previous study [33].

### 2.4. Measurement of 5-mC and 5-hmC Levels

The global DNA methylation levels for 5-methylcytosine (5-mC) and 5-hydroxymethylcytosine (5-hmC) were conducted by using commercial ELISA kits (D5425 and D5426, Zymo Research, Taipei, Taiwan). Initially, the genomic DNA was isolated from fishtail fin clips by following the instruction provided by Easy tissue and cell genomic DNA purification kit (DP021E-150, GeneMark, Taipei, Taiwan). Later, about 100 ng of isolated genomic DNA was applied to perform ELISA to quantify 5-mC and 5-hmC levels by using antigen-specific antibodies immobilized onto 96-well plates. After the color development reaction, the ELISA plate was measured by an ELISA reader (ThermoFisher Scientific, Waltham, MA, USA) at 405 nm and the concentration of 5-mC and 5-hmC was calibrated by using a standard curve according to the protocol provided by the commercial kit.

### 2.5. Multiple Behavior Test

A novel tank, mirror biting, predator avoidance, social interaction, shoaling, and circadian rhythm locomotor activity tests were performed according to our previously published methods [34,35]. In these tests, a trapezoid test tank with 22 cm along the bottom, 28 cm at the top, 15.2 cm high, and 15.9 cm along the diagonal side was used during the experiment after being filled with ≈1.25 L of filtered water. Novel tank test is a test for evaluating fish anxiety levels in the new environment. Based on the natural tendency for zebrafish to seek protection by diving, freezing, and spending the majority of time at the bottom when introduced into a novel environment, fish gradually acclimated over time, increasing in expanding their swimming area to higher portions of the test tank [36,37]. Generally, when zebrafish demonstrate anxious behavior, they tend to spend more time at the bottom of the area. Meanwhile, the mirror biting test is an assay to evaluate the aggressiveness of zebrafish. When zebrafish are introduced into a tank with a mirror attached, fish immediately display mirror biting behavior to drive away from the potential intruder. Next, the predator avoidance test is a test for evaluating the fish’s fear and escape response as innate responses to reduce the chance of being captured by the predator. When zebrafish contact with a predator, which in the current study was *Amatitlania nigrofasciata*, they display high anxiety, an elevation of serum cortisol levels, or freezing behavior [38,39]. Social interaction and shoaling tests were conducted to evaluate the social interaction between two or multiple fish. In the social interaction test, normally, zebrafish display sociality between either male–male or male–female conspecific individuals while the shoaling test was used to observe the zebrafish capability to form a shoal. Circadian rhythm locomotor activity, which reflects zebrafish circadian rhythm pattern, was also measured in the current study by monitoring their locomotor activity for 24 h [35]. In this test, six custom-made small fish tanks (20 × 10 × 5 cm) which were placed above a lightbox were used. In addition, the color preference test was also performed according to our recently published method reported by Siregar et al. [40]. This test was conducted in a 21 × 21 × 10 cm acrylic tank filled with ≈1.5 L of filtered water. Two-color combinations among red, green, blue, and yellow colors were applied in each test tank. The color perception was conducted to investigate whether the *dnmt3aa* and *dnmt3ab* KO fish exhibited any vision alteration.

### 2.6. Short-Term Memory Test (Passive Avoidance Task)

The passive avoidance task is a performance test based on fear conditioning and classically used to examine short-term or long-term memory on small laboratory animals (rats, mice, fish) [41,42,43]. In this test, a subject learns to escape an unpleasant stimulus (such as an electrical shock). Since previous studies showed that DNA methylation in the brain regulates learning and memory processes [44], we evaluated the short-term memory in *dnmt3a* KO zebrafish. We used an experimental tank (20 × 20 × 20 cm) that was divided into two chambers (bright and dark) by a separator. The zebrafish was placed in the bright chamber, and then the separator was removed. The crossing time to the dark chamber (latency) was recorded for up to 300 s. A mild electrical shock was used to punish zebrafish when it crossed into the dark chamber. Three repeated trials of training were conducted to build up the ability of zebrafish to learn and remember. The short-term memory test by passive avoidances was performed according to our previously published method reported by Bui et al. [45].

### 2.7. Video Tracking and Data Analysis

The tracked videos were recorded using open source software, idTracker that converts the fish movement data to trajectories as previously described [46]. The X and Y coordinates obtained from idTracker were then processed to obtain multiple behavioral endpoints by following our previous publication reported by Audira et al. [47].

### 2.8. Total Protein Extraction from Tissues

After the behavioral analysis, fish were randomly collected for biochemical assays. Brain and whole-body tissues were removed and a pool of three zebrafish tissues was used for homogenate preparation. Tissues were homogenized at medium speed with Bullet blender tissue homogenizer with 50 volumes of (*v*/*w*) ice-cold phosphate buffered saline (PBS), pH 7.2. Samples were further centrifuged at 12,000 g for 15 min and the crude homogenates were stored in 100 uL aliquots at −80 °C until further use. Tissue homogenates were also analyzed at the end of the behavioral experiment to observe the changes in neurotransmitters, oxidative stress, lipid peroxidation, and antioxidant activity levels. Ten biological replicates and three technical replicates were used in the analysis.

### 2.9. Determination of Neurotransmitter Contents in the Brain

The relative contents of the neurotransmitters like dopamine, GABA, serotonin, acetylcholine, glutamate, glycine, and histamine were measured using commercial target-specific ELISA kits (ZGB-E1573, ZGB-E1574, ZGB-E1572, ZGB-E1585, ZGB-E1588, ZGB-E1587, ZGB-E1586, Zgenebio Inc., Taipei, Taiwan). The oxidative stress marker (ROS), stress hormones (cortisol, catecholamine, norepinephrine, and epinephrine), and sleeping controlling hormone (melatonin) were also measured using commercial target-specific ELISA kits (ZGB-E1561, ZGB-E1575, ZGB-E1590, ZGB-E1571, ZGB-E1589, ZGB-E1597, Zgenebio Inc., Taipei, Taiwan). Initially, zebrafish tissues were minced and completely homogenized in PBS solution by using a tissue homogenizer. The target protein content of each sample was calibrated by interpolation from the standard calibration curve and normalized to the amount of total protein (μg) in each sample. The target protein content or activity was measured by following the manufacturer’s instructions.

### 2.10. Library Preparation and Whole-Genome Bisulfite Sequencing

Before bisulfite treatment, 25 ng lambda DNA was added to 5 µg fish genomic DNA. The mixed DNA was then sonicated to 450 bp and edited to blunt ending by 3′-end adenylation. Indexed paired-end adapters were added according to the manufacturer’s instructions by the Paired-End DNA Sample Prep Kit (Illumina, San Diego, CA, USA). The bisulfite conversion PCR and amplification were carried out before sequencing. Ultra-high-throughput sequencing was performed by Illumina HiSeq 4000 according to the manufacturer instructions at Genewiz Co., Ltd. (Suzhou, China). Data analyses were performed by Genewiz Co., Ltd. by using a standardized computational mapping approach to analyze the methylome.

### 2.11. DNA Extraction

Genomic DNA samples were isolated from zebrafish by using QIAamp Fast DNA Tissue Kit (QIAGEN, Hilden, Germany) according to the manufacturer’s instructions. Genomic DNA was quantified by NanoDrop 2000 (ThermoFisher Scientific, Waltham, MA, USA). High-quality DNA samples (OD 260/280 = 1.8–2.0, > 6 µg) were used to conduct the following profiling.

### 2.12. Statistical Analysis

All statistical analyses were plotted and compiled by using GraphPad Prism (GraphPad Software version 7 Inc., La Jolla, CA, USA). Before statistical analyses were conducted, data distribution normality tests were conducted to determine the statistical analysis used in each test. For behavioral tests, every fish group was compared to the wildtype zebrafish group by using different statistical analyses for each behavioral test which depended on its format and data distribution normality. Two-way ANOVA test with Geisser-greenhouse correction was used to analyze the novel tank test results while one-way ANOVA followed by Tukey post hoc test was utilized to analyze the color preference test. Meanwhile, for other behavioral tests, Mann–Whitney test was used to find the statistical significance in the distribution ranks among the groups. For the short-term memory tests, each fish group was compared using a two-way ANOVA test followed by Tukey post hoc test. Lastly, one-way ANOVA followed by Fisher’s LSD post hoc test was used to analyze the Global DNA methylation and biochemical data. The statistic details for each behavioral test are summarized in Table A1.

## 3. Results

### 3.1. Morphometric Analysis and Detection of Global DNA Methylation Levels in dnmt3aa and dnmt3ab Mutants

**Figure 2:**
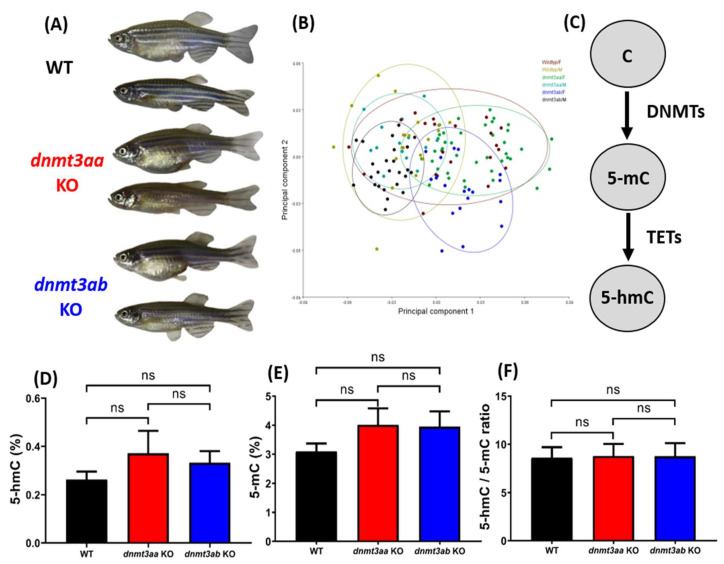
Morphometric and DNA methylation level comparison for wild types, *dnmt3aa*, and *dnmt3ab* gene knockout zebrafish. (**A**) Morphologies of six-month-old wild types, *dnmt3aa*, and *dnmt3ab* knockout zebrafish. Female fish is shown in the upper panel and male fish in the bottom panel. (**B**) Morphometric analysis of wild types, *dnmt3aa*, and *dnmt3ab* gene knockout zebrafish by principal component analysis (PCA) method. (**C**) Schematic diagram showing the biochemical pathway on key proteins involving cytosine DNA methylation. Comparison of 5-hmC (**D**), 5-mC (**E**), and 5-hmC/5-mC ratio (**F**) between wild types, *dnmt3aa*, and *dnmt3ab* knockout zebrafish. The data are expressed as the mean with S.E.M. and analyzed by one-way ANOVA followed by Fisher’s LSD post hoc test (*n* = 6 for wild type; *n* = 4 for *dnmt3aa* KO fish; *n* = 5 for *dnmt3ab* KO fish).

From the morphometric analysis results, we found that the homozygotic mutants carrying either *dnmt3aa* or *dnmt3ab* gene deficiency were viable and displayed no significant difference in their outlook to the WT zebrafish (Figure 2A,B). Afterward, the relative content of 5-mC (5-methylcytosine), 5-hmC (5-hydroxymethylcytosine), and the 5-hmC/5-mC ratio was measured to detect the Global DNA methylation. Surprisingly, based on the results obtained from ELISA, 5-mC (Figure 2E), 5-hmC (Figure 2D), and 5-hmC/5-mC ratio (Figure 2F) displayed no significant difference in either *dnmt3aa* or *dnmt3ab* KO fish lines. This result rejected our previous hypothesis and clearly demonstrates the global DNA methylation content is largely unaltered in either *dnmt3aa* or *dnmt3ab* zebrafish mutants.

### 3.2. Effects of dnmt3a Gene-Deficient on Zebrafish Locomotor Activity and Exploratory Behavior in Novel Tank Assay

**Figure 3:**
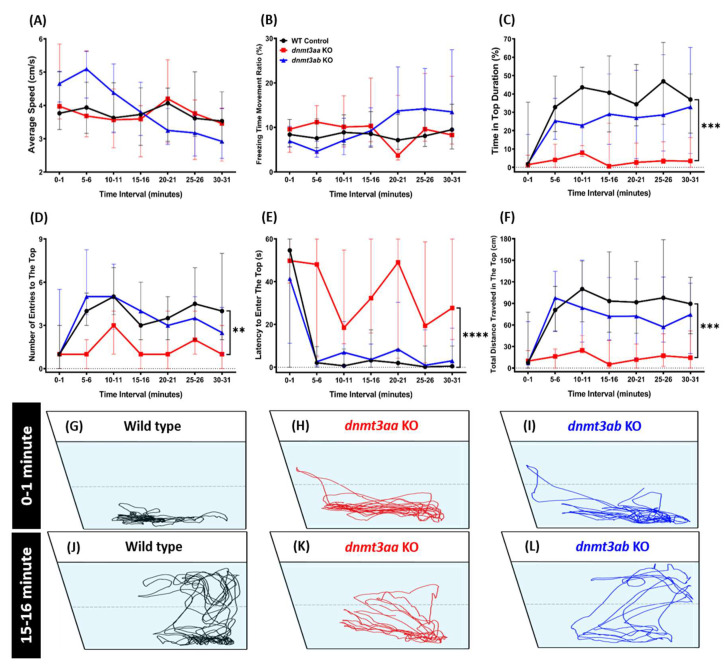
Comparison of behavior endpoints among the wild-type, *dnmt3aa,* and *dnmt3ab* mutant fish during a 30-min novel tank exploration test. (**A**) Average speed, (**B**) freezing time movement ratio, (**C**) time in bottom duration, (**D**) number of entries to the top, (**E**) latency to enter the top, and (**F**) total distance traveled in the top fish tank were analyzed. The data are expressed as the median with interquartile range and analyzed by two-way ANOVA (*n* = 30 for wild type; *n* = 7 for *dnmt3aa* KO fish; *n* = 30 for *dnmt3ab* KO fish; ** *p* < 0.01, *** *p* < 0.005, ****, *p* < 0.0001). (**G**–**I**) The locomotion trajectories of a single fish of wild type, *dnmt3aa* KO, and *dnmt3ab* KO fish, right after introduced to a novel environment. (**J**–**L**) The locomotion trajectories of a single fish of the wild type, *dnmt3aa* KO, and *dnmt3ab* KO fish after 15 min of novel tank exposure.

Overall, all of the mutant fish exhibited a quite similar level of locomotor activity compared to the control fish in the novel tank test. However, slightly more unstable locomotion activities were shown by fish with a loss function of *dnmt3ab* function. In this mutant fish, a higher average speed in the first 15 min and a lower speed with a high freezing time movement ratio afterward were observed (Figure 3A,B). Meanwhile, a similar pattern of locomotor activity with control fish was displayed by *dnmt3aa* KO fish during the whole section of the novel tank test (Figure 3A,B). However, we found that deficiency of *dnmt3aa* in zebrafish altered their exploratory behavior, which was shown by less time spent in the top area, the number of entries to the top, total distance traveled in the top, and a longer latency to enter the top portion of the tank (Figure 3C–F). Interestingly, these behavioral alterations were not found in the *dnmt3ab* KO fish (Figure 3C–F). Taken together, these observations suggested that the *dnmt3aa* KO fish may have a more severe anxious phenotype when exposed to a novel environment compared to the *dnmt3ab* mutant. The swimming trajectories for the *dnmt3aa* and the *dnmt3ab* KO fish are summarized in Figure 3G–L, and the tapped video of the behavior is included in Video S1.

### 3.3. Effects of dnmt3a Gene-Deficient on Zebrafish Aggressiveness in Mirror Biting Test

**Figure 4:**
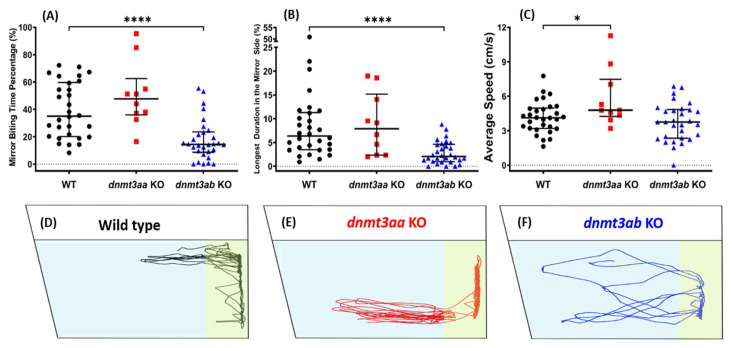
Comparison of mirror biting behavior endpoints among wild-type, *dnmt3aa*, and *dnmt3ab* mutant fish. (**A**) Mirror biting time percentage, (**B**) longest duration in the mirror side, and (**C**) average speed were analyzed. The data are expressed as the median with interquartile range and analyzed by Mann–Whitney test (*n* = 30 for wild type; *n* = 10 for *dnmt3aa* KO fish; *n* = 30 for *dnmt3ab* KO fish; * *p* < 0.05, **** *p* < 0.0001). (**D**–**F**) The locomotion trajectories of a single fish of wild type, *dnmt3aa* KO, and *dnmt3ab* KO fish, respectively, during the mirror biting test. The mirrors were put on the right side of the fish tank and the mirror biting zones were highlighted with yellow color.

**Figure A1:**
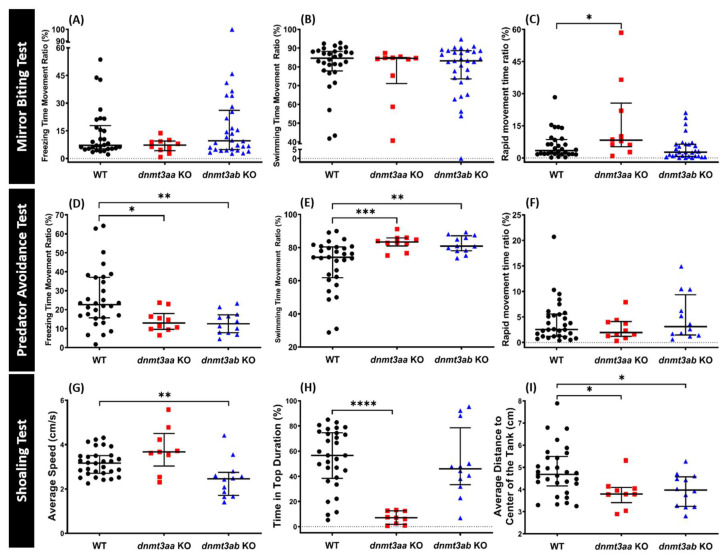
Comparison of mirror biting, predator avoidance, and shoaling endpoints among the WT, *dnmt3aa* KO, and *dnmt3ab* KO mutant fish. (**A**,**D**) Freezing time movement ratio, (**B**,**E**) swimming time movement ratio, and (**C**,**F**) rapid movement time ratio measured in mirror biting (*n* = 30 for wild type and *dnmt3ab* KO fish; *n* = 10 for *dnmt3aa* KO fish) and predator avoidance (*n* = 30 for wild type; *n* = 10 for *dnmt3aa* KO fish; *n* = 12 for *dnmt3ab* KO fish) tests, respectively. (**G**) Average speed, (**H**) time in top duration, and (**I**) average distance to the center of the tank measured in the shoaling test (*n* = 30 for wild type; *n* = 9 for *dnmt3aa* KO fish; *n* = 12 for *dnmt3ab* KO fish). The data are expressed as the median with interquartile range and analyzed by Mann–Whitney test (* *p* < 0.05, ** *p* < 0.01, *** *p* < 0.001, ****, *p* < 0.0001).

A reduced aggressive behavioral response was observed in the *dnmt3ab* KO fish (Figure 4A,B). Interestingly, the aggressiveness of the *dnmt3aa* KO fish remained unaltered (Figure 4A,B). In addition, higher average speed and rapid movement were detected in the *dnmt3aa* KO fish (Figure 4C, Figure A1C). Meanwhile, both ratios of freezing time movement and swimming time movement showed no difference between the wild type and mutant fish (Figure A1A,B). Together, we suggested that the *dnmt3ab* KO fish might have a more pronounced loss of aggression phenotype, compared to the *dnmt3aa* mutant. The mirror biting behavioral trajectories for the *dnmt3aa* and the *dnmt3ab* KO fish are summarized in Figure 4D–F, and the tapped video of the mirror biting behavior is in Video S2.

### 3.4. Effects of dnmt3a Gene-Deficient on Zebrafish Predator Avoidance Behavior

**Figure 5:**
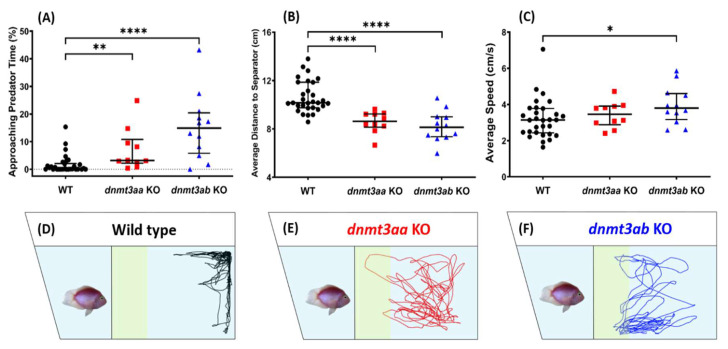
Comparison of predator avoidance behavioral endpoints among the wild-type, *dnmt3aa*, and *dnmt3ab* KO mutant fish. (**A**) Approaching predator time percentage, (**B**) average distance to the separator, and (**C**) average speed were analyzed. The data are expressed as the median with interquartile range and analyzed by Mann–Whitney test (*n* = 30 for wild type; *n* = 10 for *dnmt3aa* KO fish; *n* = 12 for *dnmt3ab* KO fish; * *p* < 0.05, ** *p* < 0.01, ****, *p* < 0.0001). (**D**–**F**) The locomotion trajectories of a single fish of wild type, *dnmt3aa* KO, and *dnmt3ab* KO fish, respectively, during the predator avoidance test. The predator fish were put on the left side of the fish tank and the approaching predator zones were highlighted with yellow color.

Next, from the predator avoidance test result, we found increments in approaching predator time and a decrease in average distance from separator in both the *dnmt3aa* and the *dnmt3ab* KO fish (Figure 5A,B). These observations suggested that loss of function on *dnmt3aa* and *dnmt3ab* activities may result in loss of fear response in zebrafish, which was considered as loss of innate life ability. Interestingly, the *dnmt3ab* KO fish demonstrated higher locomotor activity than control fish, which was shown by a significantly high level of average speed and swimming time movement ratio, and low level of freezing time movement ratio (Figure 5C, Figure A1D,E). Nonetheless, there were no differences in their rapid movement time ratio (Figure A1F). The predator avoidance behavioral trajectories for the *dnmt3aa* and the *dnmt3ab* KO fish are summarized in Figure 5D–F. The video of the predator avoidance test for all of the groups can be found in Video S3.

### 3.5. Effects of dnmt3a Gene-Deficient on Zebrafish Social Interaction

**Figure 6:**
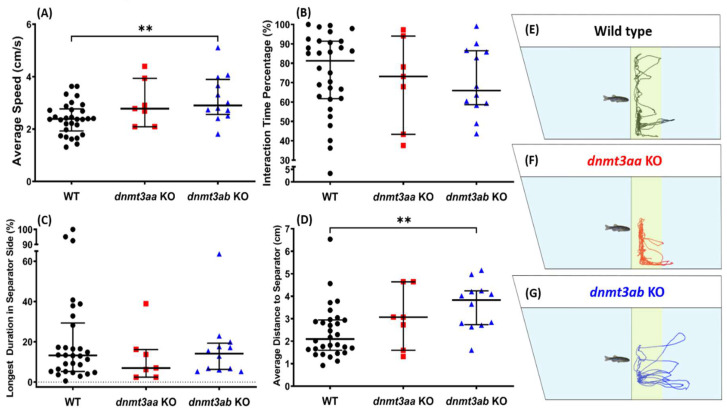
Comparison of conspecific behavior endpoints among the wild-type, *dnmt3aa*, and *dnmt3ab* mutant fish. (**A**) Average speed, (**B**) interaction time percentage, (**C**) longest duration in separator side, and (**D**) average distance to separator were analyzed. The data are expressed as the median with interquartile range and analyzed by Mann–Whitney test (*n* = 30 for wild type; *n* = 7 for *dnmt3aa* KO fish; *n* = 12 for *dnmt3ab* KO fish; ** *p* < 0.01). (**E**–**G**) The locomotion curve trajectories of a single fish of the wild type, *dnmt3aa* KO, and the *dnmt3ab* KO fish, respectively, during the social interaction test. The conspecific interaction zones were highlighted with yellow color.

Overall, in the social interaction test, either *dnmt3aa* or *dnmt3ab* KO fish did not display a pronounced social behavior alteration (Figure 6B,C). However, after further observations were conducted, a slight aberrant social behavior occurred in *dnmt3ab* KO fish, indicated with a significantly high level of the average distance to the conspecific separator (Figure 6D). Meanwhile, there was no difference in locomotion activity between *dnmt3aa* KO mutant and wild type fish, yet, a slight increase of locomotion activity was observed in the *dnmt3ab* KO mutant fish (Figure 6A). The social interaction behavioral trajectories for the control, *dnmt3aa* KO, and the *dnmt3ab* KO fish are summarized in Figure 6E–G, and the video for the social interaction behavior can found in Video S4.

### 3.6. Effects of dnmt3a Gene-Deficient on Zebrafish Shoaling Formation

**Figure 7:**
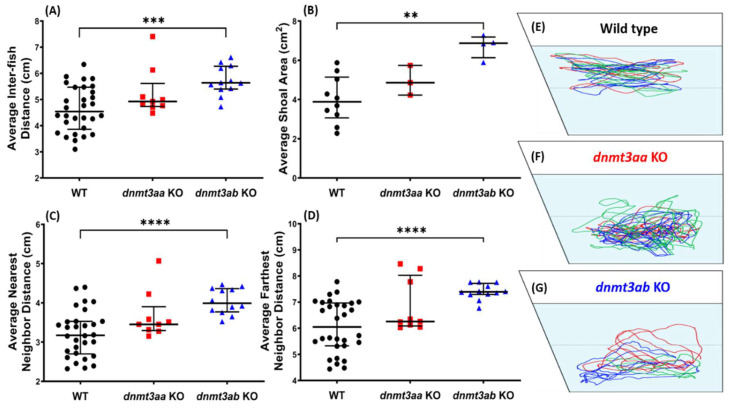
Comparison of shoaling behavior endpoints among the wild-type, the *dnmt3aa*, and the *dnmt3ab* mutant fish. (**A**) Average inter-fish distance, (**B**) average shoal area, and (**C**) average nearest neighbor distance, and (**D**) average nearest neighbor distance were analyzed. Three fish were used in one group (shoal) for the shoaling test. The data are expressed as the median with interquartile range and analyzed by Mann–Whitney test (*n* = 30 for wild type; *n* = 9 for *dnmt3aa* KO fish; *n* = 12 for *dnmt3ab* KO fish; ** *p* < 0.01, *** *p* < 0.001, ****, *p* < 0.0001). (**E**–**G**) The locomotion trajectories of three fish of the wild type, the *dnmt3aa* KO, and the *dnmt3ab* KO fish, respectively, during the shoaling test.

Shoaling test, another social behavior test to evaluate socializing, showed the associated effect caused by *dnmt3ab* deficiency. Significantly high levels of average inter-fish distance, shoal area, nearest neighbor distance, and farthest neighbor distance were shown in the *dnmt3ab* mutant group (Figure 7A–D). On the other hand, zebrafish with a deficiency in *dnmt3aa* exhibited similar shoaling behavior to the control group (Figure 7A–D). However, consistent with the novel tank test result, loss of exploratory behavior was displayed by *dnmt3aa* KO mutant fish. This phenomenon was indicated by significantly low levels of time in top duration and slightly lower average distance to the center of the tank (Figure A1H,I). Interestingly, a less pronounced exploratory behavior was also observed in the *dnmt3ab* KO mutant fish, which was also shown by a slightly lower average distance to the center of the tank, which may be related to the low locomotion activity exhibited by the mutant fish during the test (Figure A1G,I). In summary, loss of function of *dnmt3ab* in adult zebrafish related to the loose shoal formed during the test. The shoaling behavioral trajectories for the WT, *dnmt3aa* KO, and the *dnmt3ab* KO fish are summarized in Figure 7E–G, and the tapped videos for the shoaling behavior of each group can be found in Video S5.

### 3.7. Effects of dnmt3a Gene-Deficient on Zebrafish Circadian Rhythm Locomotor Activity

**Figure 8:**
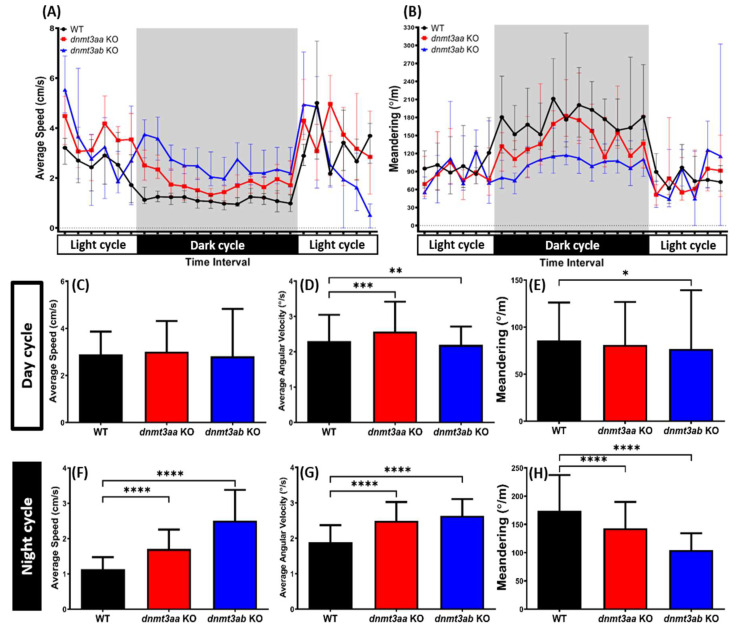
The circadian rhythm locomotor activity for wild-type, *dnmt3aa*, and *dnmt3ab* mutant fish. (**A**,**B**) Comparison of the average speed and meandering, respectively, between wild-type, *dnmt3aa*, and *dnmt3ab* KO fish during the light and dark cycles. Comparisons of the average speed (**C**,**F**), average angular velocity (**D**,**G**), and meandering (**E**,**H**) in light and dark cycles, respectively, were demonstrated. Data are presented as median with interquartile range and analyzed by Mann–Whitney test (*n* = 28 for wild type; *n* = 18 for *dnmt3aa* KO fish; *n* = 18 for *dnmt3ab* KO fish; * *p* < 0.05, ** *p* < 0.01, *** *p* < 0.005, **** *p* < 0.0001).

During circadian rhythm locomotor activity test with a light/dark (12/12) condition, we observed that control fish and the mutant fish showed different speed and meandering patterns in most of the time intervals, suggesting that loss of function of the *dnmt3a* gene in the zebrafish caused an irregular pattern of circadian rhythm locomotor activity (Figure 8A,B). Interestingly, after further examination, both *dnmt3a* KO zebrafish were found to maintain a similar level of average speed during the daytime interval compared to the control fish (Figure 8C). However, irregular movement of zebrafish, indicating by the abnormalities in average angular velocity and meandering, was observed in both KO fishes. A higher average angular velocity was exhibited by *dnmt3aa* KO mutant fish while a low angular velocity was seen in the *dnmt3ab* KO mutant fish during the day cycle (Figure 8D,E). Furthermore, hyperactivity-like behavior during the night cycle was observed in both KO fishes (Figure 8F–H). Video for circadian rhythm locomotor activity behavior can be found in Video S6.

### 3.8. Effects of dnmt3a Gene-Deficient on Zebrafish Color Preference Ranking

**Figure 9:**
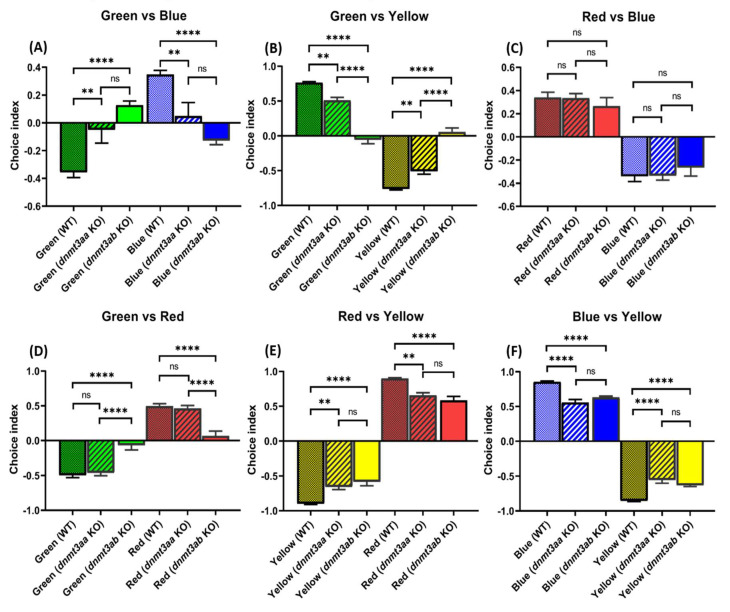
Comparison of color preference ranking and index between wild type, *dnmt3aa* KO, and *dnmt3ab* KO mutant zebrafish: (**A**) green vs. blue combination; (**B**) green vs. yellow combination; (**C**) red vs. blue combination; (**D**) green vs. red combination; (**E**) red vs. yellow combination; and (**F**) blue vs. yellow combination. Data were analyzed with one-way ANOVA followed by Tukey post-hoc test. The data were presented as mean ± S.E.M. (*n* = 24, ** *p* < 0.01, **** *p* < 0.0001).

We found that the color preference patterns of the *dnmt3aa* and *dnmt3ab* KO fish were more diverse compared to the wild type. While the wild type fish showed color preference ranking as red > blue > green > yellow, the *dnmt3aa* KO fish showed altered color preference ranking as red > blue = green > yellow, and *dnmt3ab* KO fish showed altered color preference as red = green > blue > yellow. Color preference between green and blue was reduced or reversed in *dnmt3a* mutants. The *dnmt3aa* KO fish did not have preferences between green or blue, while *dnmt3ab* KO fish switched their preferences from blue to green preferences (Figure 9A). In addition, the *dnmt3aa* KO fish showed the same preference pattern as the wild type, yet, the *dnmt3ab* KO fish showed no preferences between green and red color (Figure 9D). All other color combinations showed a significant decrease in choice index value for the *dnmt3aa* and the *dnmt3ab* KO fish. The green-yellow combination showed a decrease in both the *dnmt3aa* and the *dnmt3ab* KO fish showed a decrease in the green-yellow preference index. Moreover, the *dnmt3ab* KO fish display the most significant reduction in green preference (Figure 9B). Lastly, although both KO fish showed less preference in red-yellow (Figure 9E) and blue-yellow (Figure 9F) combination, no significant difference was found.

### 3.9. Effects of dnmt3a Gene-Deficient Zebrafish on Short-Term Memory

**Figure 10:**
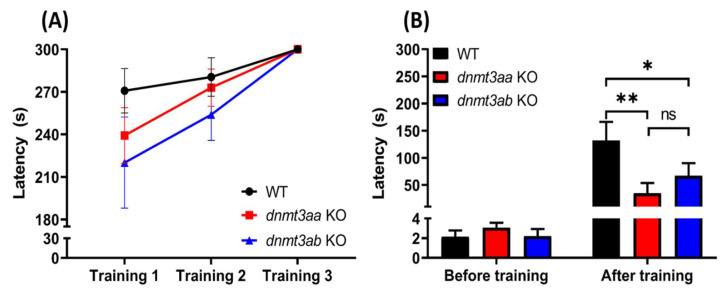
Comparison of short-term memory between wild type, *dnmt3aa* KO, and *dnmt3ab* KO mutant zebrafish. (**A**) The latency of fish swimming into the dark chamber after electrical shock was given in three training sessions. (**B**) The latency of fish swimming into the dark chamber one day after training sessions. Data were analyzed with two-way ANOVA followed by Tukey post hoc test. The data are presented with mean ± S.E.M. (*n* = 15 for WT and *dnmt3ab* KO fish; *n* = 16 for *dnmt3aa* KO fish, * *p* < 0.05, ** *p* < 0.01).

In short-term memory test, we observed the latency on all zebrafish groups was increased along with trials in the training session but was not significantly longer (Figure 10A). Additionally, no significant difference can be found in the latency during first to third training between wild type, *dnmt3aa* KO, and *dnmt3ab* KO zebrafish. However, the *dnmt3aa* and *dnmt3ab* mutant zebrafish displayed significantly lower memory retention with a reduction of the latency down to below 100s in one day after the training session (Figure 10B). This result demonstrated that the loss of *dnmt3a* function can induce short-term memory loss.

### 3.10. Biochemical Assay of dnmt3a Gene-Deficient Zebrafish

By using ELISA (enzyme-linked immunosorbent assay), the relative content of several important neurotransmitters (like dopamine, GABA, serotonin, norepinephrine, acetylcholine, glutamate, glycine, histamine, catecholamine, and epinephrine) and other biomarkers (ROS, cortisol, melatonin) in the fish brain or whole-body tissues were measured. To our surprise, ELISA quantification results in zebrafish brain tissue showed no significant difference in the neurotransmitter levels between control and mutant fish brains (Table 1). Later, to better explore the potential mechanism, we also measured the neurotransmitter contents in the whole-body tissue. Neurotransmitters, such as acetylcholine, catecholamine, and epinephrine were higher within the body area of *dnmt3ab* KO fish and lower in the *dnmt3aa* KO fish compared to those in the wild type fish (Table 2). Acetylcholine, a neurotransmitter that releases signals to adjacent motor neurons, activates the skeletal muscle and causes contraction [48]. It is associated with physiological and behavioral processes in central neuron system (CNS) during cholinergic signaling [49]. A previous study proved that acetylcholine in the brain altered neuronal excitability and modified brain response to internal and external inputs [48]. In our study, differences in acetylcholine level altered innate behaviors in *dnmt3aa* and *dnmt3ab* zebrafish. Both catecholamine and epinephrine would be increased in stressful conditions [50]. The reactive oxygen species (ROS) level was also higher in the *dnmt3ab* and lower in the *dnmt3aa* compared to that in wild type fish, suggesting the ROS scavenging capacity in the *dnmt3ab* KO fish was somehow attenuated (Table 2). ROS has been associated with oxidative stress and signaling stress response [51]. ROS is also known for its effect on neuronal death and neurological defect that correlated with behavior alteration [52]. These data might explain the aberrantly anxious behavior, especially in the *dnmt3ab* KO fish. However, in the *dnmt3aa* KO fish, all the detected neurotransmitters in the fish body showed less expression than that in the wild type fish; the *dnmt3aa* fish only showed a slight loss of exploratory ability and minor dysregulation of the circadian rhythm locomotor activity. Taken together, these results emphasized that the deficiency in *dnmt3ab* is more dominant to fish; however, gene expression profiling will help to unveil the rationale of disparate modulation of emotion and social interaction.

### 3.11. Profiling of Genome-Wide DNA Methylation Sequencing

Since no difference in methylation level was detected by measuring the 5-hmC/5-mC ratio, we performed whole-genome methylation sequencing to get better resolution. To elucidate the genome-wide methylation status, genomic DNA isolated from wild type AB strain (WT), *dnmt3aa*, and *dnmt3ab* KO zebrafish brain tissues were subjected to construct genomic library to perform bisulfite deep sequencing. In total, 332,343,888, 323,975,024, and 332,003,988 bisulfite deep sequencing reads by paired-end sequencing were obtained from WT, *dnmt3aa*, and *dnmt3ab* KO zebrafish, respectively. Of the raw reads from each sample, 75.34% (250,402,950), 74.83% (242,424,388), and 74.70% (248,000,732), respectively, can be successfully mapped back to the reference genome, while of the 85.87% (215,030,868), 85.60% (207,507,370), and 86.01% (213,315,676) of the mapped reads were uniquely mapped to the reference genome, respectively. The coverage of sequencing data was 84.61%, 84.19%, and 85.08% for each sample. The CG percentages were 19.66%, 19.94%, and 19.50%, respectively. The mean depths were 25.69, 24.88, and 25.38, which were sufficient for high-quality genome-wide methylation analysis. Following the bisulfite sequencing to analyze the genome-wide methylated cytosines (mC), a total of 39,055,549, 38,502,276, and 39,404,575 mC were counted for wild type, *dnmt3aa*, and *dnmt3ab* mutants, respectively. The percentages of CG methylation in total CG number in WT, *dnmt3aa*, and *dnmt3ab* samples were 77.01%, 76.32%, and 77.20%, respectively. Not only the total count of mC, but also each type of methylation, including mCG, mCGH, and mCHH (H = A, T, or G) showed no significant difference between groups. The distribution of bases near mC sites and the probability of methylated types were calculated between 9 bp bases by using WebLogo (http://weblogo.berkeley.edu/logo.cgi). Among the methylation sites, the complete distribution on zebrafish chromosomes are listed in Tables S1–S3 for WT vs. *dnmt3aa* KO, WT vs. *dnmt3ab* KO, and *dnmt3aa* KO vs. *dnmt3ab* KO, respectively. Eight functional regions were divided based on gene structures, including exonic, intergenic, intronic, splicing, upstream, downstream, 3′ untranslated region (UTR3), and 5′ untranslated region (UTR5) regions. The term of upstream and downstream represents 1000 bp from the coding gene. Among these regions, the intronic region revealed the highest methylation level in all three groups (summarized in Table 3).

### 3.12. Identification of Differentially Methylated Regions (DMR) and Functional Analysis of DMR-Associated Genes

**Figure 11:**
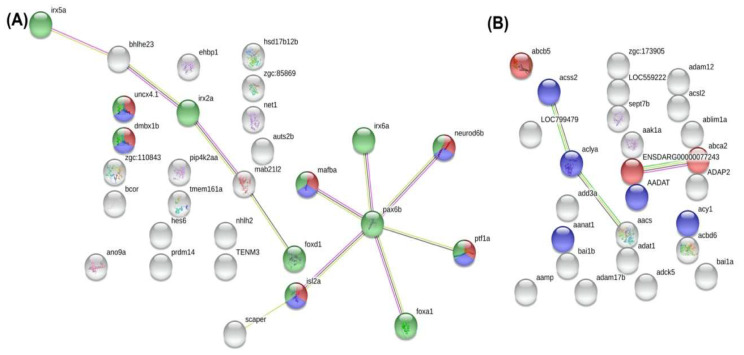
STRING protein interaction networking analysis between the (**A**) WT and *dnmt3aa*, and (**B**) WT and *dnmt3ab* KO zebrafish gene sets. (**A**) Red node represented protein involving in neuronal development; blue node represented DNA binding proteins; green node represented nucleic proteins. (**B**) Red node represented ABC transporter-related proteins; blue node represented proteins that associated metabolic pathways.

To address the effects of *dnmt3a* mutants on the methylation level, the differentially methylated regions (DMRs) between three groups were analyzed. A total of comparison between WT vs. *dnmt3aa*, WT vs. *dnmt3ab*, and *dnmt3aa* vs. *dnmt3ab* are listed in Table 3. We found that wild type zebrafish showed more DMRs between *dnmt3aa* KO (15962 sites), compared to *dnmt3ab KO* (9543 sites). Among all DMRs, a threshold was set at > ±0.8 in β value difference (delta β value) as hyper-methylated, while < ±0.2 in β value as hypo-methylated. Additionally, 5103 (10 hyper and 5097 hypo), 1912 (2 hyper and 1910 hypo), and 806 (561 hyper and 245 hypo), 3658 (12 hyper and 3646 hypo) DMRs were identified in three comparisons, respectively. On the other hand, the top 30 genes that consisted of most DMRs are listed in Tables S1–S3. To evaluate the genes affected by *dnmt3a* mutations, annotated pathways for the top 30 DMR sites between groups were predicted with The Database for Annotation, Visualization, and Integrated Discovery (DAVID) (Table S4). Among these, pathways with *p* < 0.05 were significantly enriched. We also input differential expression genes into STRING to analyze the potential protein–protein interaction networks (Figure 11). Within the genes that significantly expressional diverse between WT and *dnmt3aa* KO fish, *neurod6b*, *ptfa1*, *mafba*, *isl2a*, *uncx4.1*, *dmbx1b*, and *mab21l2* encoded DNA-binding proteins that involved in neural development (Figure 11A). Meanwhile, the genes that significantly expressional diverse between WT and *dnmt3ab* KO fish, including ENSDARP00000099145 (*abcc3*), *abca2*, and *abcb5*, were reported to relate ATP-binding cassette (ABC) transporter pathway. On the other hand, *aadat*, *acy1*, *acss2*, *aclya*, and *aanat1* were associated with various metabolic pathways, which involved in acetyl-CoA synthesis and aminotransferase (Figure 11B). We concluded those DMRs identified by whole genome methylation sequencing might be associated with behavioral alteration in *dnmt3aa* or *dnmt3ab* mutants and provide a good entry point for functional validation in the future.

## 4. Discussion

### 4.1. Novel and Non-Overlapped Functions of dnmt3aa and dnmt3ab Genes on Modulating Behaviors in Zebrafish

In this study, two zebrafish mutant lines carrying either *dnmt3aa* or *dnmt3ab* gene deficiency were established by TALEN genome editing tool and reported having different behavioral alterations for the first time. Previous studies demonstrated that in rat models, dysregulation of DNA methylation is related to anxiety and major depressive disorder [14,15,16,17]. Furthermore, mice that lack *Dnmt* exhibited abnormal hippocampal CA1 long-term plasticity and deficits of learning and memory [53]. By multiple behavior assay, *dnmt3aa* KO fish were noticed with exploratory behavior, predator avoidance, and sleep behavioral alterations. Meanwhile, alterations in aggressiveness, predator avoidance, social interaction, shoaling formation, sleep behavior, and color preference index ranking was detected in the *dnmt3ab* KO fish. These findings provided in vivo and direct evidence on supporting that *dnmt3aa* and *dnmt3ab* genes play important and non-overlapping roles in modulating behavior in zebrafish for the first time. In addition, since the aim of the current study was to study the functions of these genes in zebrafish behavior in general, one has to keep in mind that this study used zebrafish of mixed gender. Additionally, this was also taken based on several prior studies in zebrafish behaviors that also used mixed-gender zebrafishes [34,54,55,56]. However, even though several studies showed that there is no gender effect in some zebrafish behavior tests [57,58,59], there are also several studies that mentioned the existence of gender effect in zebrafish behavior tests [60,61,62]. Thus, there is still a possibility that the gender effect in these behavioral tests might be possessed by the mutant fishes, which is worth trying in future studies.

### 4.2. The dnmt3a Gene Contributes to Zebrafish Behavior Responses to a New Environment

In our study, abnormal locomotor activity pattern after novel environment exposure was detected in *dnmt3ab* KO fish. Altered catecholamine (epinephrine and norepinephrine) and acetylcholine levels in these mutant fish may be related to their abnormal behavior. In all vertebrate species, catecholamines are released into the general circulation that is required to enhance blood oxygen transport and the mobilization of energy substrates. Therefore, the release of catecholamines is an integral part of the physiological response to stressors in all vertebrate groups [63]. Supporting the result of this study, a previous study in the rainbow trout (*Oncorhynchus mykiss*) found that exposure to carbamate pesticides altered catecholamine levels and affected its neurotransmitters and behavior [64]. In addition, another previous study found that the administration of polychlorinated biphenyls in the killifish (*Fundulus grandis*) altered brain levels of dopamine and norepinephrine and affected locomotor activity [64]. Furthermore, an abnormal level of acetylcholine (ACh), a neurotransmitter at a synaptic junction, may also play a role in the locomotion behavior alteration of fish due to inhibition of acetylcholinesterase (AChE) activity in the brain. A prior study found that sublethal exposure of chlorpyrifos, an organophosphorus insecticide that elicits toxicity through inhibition of AChE enzyme, in mosquitofish (*Gambusia affinis*) caused a stressful condition and reduced their locomotion behavior [65,66]. Next, exploratory behavior deficits showed by *dnmt3aa* KO fish may also be related to altered levels of catecholamine found in this study. Homozygous deletion on COMT (catechol-O-methyltransferase), one of the major mammalian enzymes involved in the metabolic degradation, impairment in emotional reactivity in the dark/ light exploratory model of anxiety were displayed in female, but not in male mice [67]. Exposure of chlorpyrifos in common carp fingerlings also caused the carp to exhibit irregular, erratic, and darting swimming movements, hyperexcitability, loss of equilibrium, and sinking to the bottom. The detected behavioral changes may be due to the abnormal level of ACh in cholinergic synapses leading to hyperstimulation and cessation of neuronal transmission (paralysis) [68].

### 4.3. The dnmt3ab Gene Contributes to Zebrafish Aggressive Behavior

Aggression is an important component of the behavioral repertoire of animals that plays a major role in their Darwinian fitness [69], which served various adaptive functions, including the establishment of dominant relationships, hierarchies, and the competition for life resources in zebrafish [26,69]. In general, aggressive behavior is regulated by hormones in the brain [70]. However, the role of *dnmt3aa* and *dnmt3ab* genes in aggression is rarely studied. In our study, we provided direct evidence that connects the *dnmt3a* gene to aggression. Our results indicated that the *dnmt3ab* KO fish showed loss of aggression, which was supported by a dysregulated level of serotonin (5-HT), one of the chemicals that is critically involved in the neural circuits for many types of human and animal aggression [71], detected in the *dnmt3ab* KO fish body. Alterations in 5-HT neurotransmission have been found in several of the KO mice that displayed unusual aggressive behavior [72]. In humans, serotonergic dysfunction that altered levels of brain 5-HT also influences aggression [73]. Furthermore, an altered level of norepinephrine (NE), one of the classic neurotransmitters found in the peripheral and central nervous system (CNS), may also contribute to behavior alteration, which regulates physiological status, including mood, learning and memory, arousal, blood flow, and metabolism [74,75]. In addition, studies from the psychiatric clinic implied that alterations of NE and 5-HT, as well as the third monoamine, dopamine (DA), were present in humans with emotional disorders [76].

### 4.4. The dnmt3aa and dnmt3ab KO Fish Behaved Boldly in the Presence of a Predator

Predator avoidance, one of the fear responses [77], is conducted to respond to danger and may allow the zebrafish to avoid dangers in nature. Here, a novel finding was shown indicating *dnmt3aa* and *dnmt3ab* play a role in the reduction of zebrafish predator avoidance response. Consistent with previous studies, serotonin and catecholamine may be involved in this behavior dysregulation. For example, dysfunction of the serotoninergic system has been associated with fear (anxiety), locomotion, feeding, asymmetry of alcohol effects, depression, stress, and aggression [78,79]. Furthermore, catecholamines generally appear to be closely related to behavioral arousal. Existing data in rats indicate that the catecholamines may be important at least during the early acquisition of learned responses motivated by fear [80]. Another study in rats also found that depletion of local catecholamines including dopamine impaired extinction of a conditioned fear response under particular conditions [81]. In addition, tyrosine, a precursor in the biosynthesis of catecholamines, has been shown to enhance fear-induced immobility when administered systemically in rats [82].

### 4.5. The dnmt3ab KO Fish Displayed Alterations in Both Social Tests

Shoaling behavior is characterized by distance within a group that would be expected in case of random spatial distribution between individuals [78]. According to our results, it was found that the loss function of *dnmt3ab* resulted in loosened shoal. The aberrant shoaling behavior may due to an increased anxiety level in the *dnmt3ab* KO fish. Furthermore, this finding is also supported by the less conspecific interaction displayed by this mutant fish in the social interaction test. These phenomena are consistent with previous studies that found the relationship between serotonin and social behavior [83]. Despite the association with aggression, dysfunction of the serotoninergic system has been known to be associated with antisocial behavior in humans. In zebrafish, a reduced level of serotonin was shown to be related to shoaling [79,84,85]. Besides, serotonergic activity has also been correlated to the social status in primates, other mammals, reptiles, and fishes [79]. Moreover, DDT (Dichloro-Diphenyl-Trichloroethane) exposure, which increased spontaneous activity and interfered with schooling behavior, elevated brain 5-HT, and decreased dopamine levels in goldfish (*Carassius auratus*) [64].

### 4.6. The dnmt3aa and dnmt3ab Genes Inducing the Abnormalities toward Circadian Rhythm Locomotor Activity

To cope with environmental cycles, fish display circadian rhythm as their response [86]. Circadian rhythm changes on a daily basis and is driven by autonomous circadian clocks. Circadian clocks affect most aspects of vertebrate physiology and behavior by generating daily cycles in sleep and alertness, body temperature, hormone secretion, metabolism, blood pressure, intraocular pressure, and visual sensitivity. However, little is known about how the *dnmt3a* gene function controls zebrafish circadian rhythm. In our study, direct evidence that links *dnmt3aa* and *dnmt3ab* gene function on modulating circadian rhythm in zebrafish was reported. As the result, altered levels of melatonin and serotonin found in the mutant fish may be connected to this phenomenon. Melatonin, an endogenous indolamine, is a well-conserved feature in vertebrates that contributes to the entrainment of daily and annual physiological rhythms [87]. Melatonin synthesis occurs in the pineal gland and in the retina. Studies on amphibians, birds, and rodent retinas indicate that melatonin synthesis exhibits circadian rhythmicity [88]. Cahill and colleagues demonstrated that the zebrafish pineal contains a self-sustaining circadian oscillator that regulates melatonin synthesis, as well as a phototransduction mechanism sufficient for the entrainment of the oscillator [89]. In addition, serotonin appeared to be necessary for slow-wave sleep norepinephrine for arousal and REM (rapid eye movement) sleep [76].

### 4.7. Distinct Preferences of dnmt3aa and dnmt3ab KO Fish toward Visual Stimuli

The changes for the color preferences can be seen as a phenotypic mutation caused by several factors that still need to be explored. Several reports have described the decrease in color preferences as vision-related mutation [90], depression-related behavior [91], dark-light preferences have been linked with the left-habenula [92], and heavy-metal exposure also has been reported to alter zebrafish color preference [93]. The color preference may be used as one of the parameters to assess anxious phenotype that was used to study the loss function of *dnmt3aa* and *dnmt3ab* effect, especially in the zebrafish color perception. The changes can be seen related to the green and blue preferences for the pattern changes. The zebrafish has been reported to prefer lower wavelength compared with higher wavelength [93,94], however, *dnmt3aa* and *dnmt3ab* mutant fish did not show that behavior. Further investigations are needed to link the behavior changes with the mutation caused by *dnmt3aa* and *dnmt3ab* gene deficiency at the molecular level.

### 4.8. dnmt3a Gene is Essential for Memory Retention in Zebrafish

The memory impairment on *dnmt3a* gene-deficient zebrafish was displayed by the passive avoidance test. This finding is consistent with the previous publication that revealed the knockout of *dnmt3a* in adult mice has deficits in hippocampus-dependent learning and memory. The deletion of *dnmt3a* also can further affect brain development with smaller hippocampi found in *dnmt3a* KO mice compared to control mice [53]. DNMT3a together with other DNMTs are important DNA methyltransferases that might target specific genes involved in gene expression, synaptic function, learning, and memory [44]. A previous study showed that DNA methylation modulates the expression of *reelin*, *calcineurin*, brain-derived neurotrophic factor (BDNF), and protein phosphatase-1 (PP1) which are associated with learning and memory [95,96,97]. Another study also showed that the restoration of the *dnmt3a* level in the adult brain of mice improved memory in fear conditioning tasks [97]. From the prior study, the deletion of *dnmt3a* also can further affect brain development with smaller hippocampi found in *dnmt3a* KO mice compared to control mice [53]. Supported by these findings, our data also found the loss or depletion of *dnmt3aa* or *dnmt3ab* could impact cognition formation resulting in the deficit of learning and memory in the mutant zebrafish.

### 4.9. The Differences in Neurotransmitters and Methylation Level of dnmt3a Gene-Deficient Zebrafish

Surprisingly, the neurotransmitters and other biomarkers in brain tissues of mutant zebrafish were relatively on a similar level with the wild type. On the contrary, several neurotransmitters from the whole body of *dnmt3a* mutant fish were altered. The changes in the mutant zebrafish might be compromised only on the epigenetic scale. DNA methylation is a common type of epigenetic modification [98]. Epigenetics is associated with phenotypic variation, including behavioral variation [99,100]. We observed phenotype changes with behavioral alteration although no biochemical variation in the brain of mutant zebrafish. Additionally, the methylation in each tissue and even in each cell is unique. Each tissue has its own methylation pattern that specifies its identity and functions [101]. That explains why the neurotransmitters and biomarkers content in the brain of *dmnt3a* KO fish could remain unaltered, while on the other hand altered the level of the neurotransmitters in the whole body. The exact mechanism of how the methylation is specified into specific cell types remains unknown. However, one thing is certain, *dnmt3a* mutations or loss lead to a differentiated phenotype [102].

Following our findings, we further measured the methylation level in *dnmt3a* mutant zebrafish. Methylation and demethylation are critical tools to regulate the neuronal methylome in the context of cognition and behavior [103]. The DNA methylation process is involved in the interaction with several elements such as hormones, transcription factors, and neurotransmitters [101]. The remaining unaltered DNA methylation level in this experiment also might have happened because we only generated zebrafish with a lack of *dnmt3a*. A previous study found only double knockout on both *dnmt1* and *dnmt3a* exhibited a decrease in DNA methylation [53]. It suggested that *dnmt1* and *dnmt3a* are DNMT members that have overlapping functions. Dnmt3a and Dnmt3b are the de novo DNMT family that contribute to establishing the DNA methylation pattern during primordial germ cell development and early embryogenesis. While, dnmt1 is responsible for the maintenance of genomic DNA methylation patterns globally [104]. A prior study showed that the disruption in *dnmt3a* and *dnmt3b* mice embryos leads to an impairment of de novo methylation during development, but it has no effect on the preexisting methylation. Conversely, the inactivation of *dnmt1* results in global demethylation [6]. Our results, therefore, demonstrated that *dnmt3a* actually plays role in methylation during early development, thus might affect behavior and cognition in the mutant zebrafish.

According to the diverse genetic pattern obtained from whole-genome methylation sequencing between WT and *dnmt3aa* KO fish, *neurod6b*, *ptfa1*, *mafba*, *isl2a*, *uncx4.1*, *dmbx1b*, and *mab21l2* were associated with neural development, which indicated that *dnmt3aa* KO affects neurons through these genes. *Neurod6*, an abbreviation of neurogenetic differentiation 6, was reported to associate with the neurodevelopmental disease, long-term potentiation, as well as synaptic transmission defects [105,106]. In addition, *ptfa1* was also considered as one of the regulators of transcriptional homeodomain during neuronal specification [107]. Not only neuronal disorder, but *pax6b* mutant zebrafish also showed eye defects, which suggested the color preference in our study may be due to aberrant Pax6b expression [108,109]. On the other hand, compared to WT and *dnmt3ab* KO fish, ABC transporter-associated network was affected. One of the ATP-binding cassettes, *Aabca7*, was considered as a novel biomarker in Alzheimer’s disease. Moreover, epigenetic markers on *Abcaa7* are also significantly related to Alzheimer’s disease [110], which provided the evidence that *dnmt3ab* may cause brain damage through ABC transporters and showed early onset of anxiety in our KO model. Another key pivot within *dnmt3ab* affected network is acetyl-CoA-associated genes, which are linked to several neurodegenerative disorders and aging [111,112].

In conclusion, this study generated two important zebrafish mutants carrying either *dnmt3aa* or *dnmt3ab* gene mutations by the TALEN-mediated genome editing tool. Similar but non-overlapped functions were identified by a battery behavioral assay between *dnmt3aa* or *dnmt3ab* mutants. We successfully demonstrated that the *dnmt3ab* mutants display very strong anxiety identified and predicted by the novel *dnmt3a*-associated gene network that is related to cognitive behaviors, although the morphometric and biochemical levels remain unaltered (summarized in Table 4). However, how those genes function associated with *dnmt3a* genes requires further examination. Additionally, a histopathology measurement, to validate the possible reason for alteration in behavior related to tissue or organ developmental, deserves further attention. Future studies to elucidate specific roles of *dnmt3aa* and *dnmt3ab* in the developmental and neurogenetic disorder could provide valuable new insights. In addition, as mentioned above, it is also intriguing to conduct another study for observing whether there is a gender-effect related to these KO fish behavior abnormalities.

## Figures and Tables

**Table 1:** Comparison of neurotransmitter and other biomarker levels in the brain of wild type (WT), *dnmt3aa* KO, and *dnmt3ab* KO zebrafish. The data are expressed as the mean with S.E.M. and analyzed by one-way ANOVA followed by Fisher’s LSD post hoc test (*n* = 10 for all groups).

Biomarkers	WT	*dnmt3aa* KO		*dnmt3ab* KO		Unit	F (DFn, DFd)	ANOVA
	Concentration	Concentration	*p-*Value	Concentration	*p-*Value			Significance
Dopamine	1.7380 ± 0.2852	1.9060 ± 0.3808	0.7410	1.7550 ± 0.3898	0.9737	pg/μg	F (2, 27) = 0.06767	NO
GABA	0.0093 ± 0.0017	0.0099 ± 0.0023	0.8345	0.0091 ± 0.0020	0.9445	nmol/μg	F (2, 27) = 0.04286	NO
Serotonin (5-HT)	0.0290 ± 0.0058	0.0331 ± 0.0087	0.7020	0.0289 ± 0.0077	0.9925	ng/μg	F (2, 27) = 0.1022	NO
Norepinephrine	0.1711 ± 0.0333	0.1749 ± 0.0379	0.9417	0.1499 ± 0.0378	0.6837	ng/μg	F (2, 27) = 0.1369	NO
ROS	0.5943 ± 0.0918	0.5890 ± 0.1273	0.9721	0.4874 ± 0.0961	0.4831	U/mL	F (2, 27) = 0.3212	NO
ACh	0.7061 ± 0.1742	0.9756 ± 0.2387	0.3193	0.8205 ± 0.1360	0.6701	ug/μg	F (2, 27) = 0.5187	NO
Cortisol	2.2670 ± 0.3324	2.2670 ± 0.3272	0.9985	1.7780 ± 0.1878	0.2445	pg/μg	F (2, 27) = 0.9451	NO
Glutamate	0.0262 ± 0.0044	0.0311 ± 0.0054	0.4975	0.0329 ± 0.0052	0.3554	ug/μg	F (2, 27) = 0.4737	NO
Glycine	0.1369 ± 0.0207	0.1788 ± 0.0240	0.1911	0.1526 ± 0.0214	0.6194	ug/μg	F (2, 27) = 0.9181	NO
Histamine	0.0170 ± 0.0049	0.0183 ± 0.0040	0.8360	0.0177 ± 0.0043	0.9112	ng/μg	F (2, 27) = 0.02188	NO
Catecholamine	1.4080 ± 0.3270	1.3790 ± 0.2444	0.9437	1.3450 ± 0.2853	0.8792	ng/μg	F (2, 27) = 0.01179	NO
Melatonin	0.0596 ± 0.0083	0.0718 ± 0.0096	0.5514	0.0782 ± 0.0213	0.3659	pg/μg	F (2, 27) = 0.4366	NO
Epinephrine	0.0091 ± 0.0014	0.0092 ± 0.0012	0.9579	0.0099 ± 0.0013	0.6733	ng/μg	F (2, 27) = 0.1079	NO

**Table 2:** Comparison of neurotransmitter and other biomarker levels in the body of wild type (WT), *dnmt3aa* KO, and *dnmt3ab* KO zebrafish. The data are expressed as the mean with S.E.M. and analyzed by one-way ANOVA followed by Fisher’s LSD post hoc test (*n* = 10 for all groups, ** *p* < 0.01, *** *p* < 0.001).

Biomarkers	WT	*dnmt3aa* KO		*dnmt3ab* KO		Unit	F (DFn, DFd)	ANOVA
	Concentration	Concentration	*p-*Value	Concentration	*p-*Value			Significance
Dopamine	0.7943 ± 0.2082	0.5660 ± 0.1293	0.3937	1.0830 ± 0.2098	0.2821	pg/μg	F (2, 27) = 1.938	NO
GABA	0.0041 ± 0.0012	0.0026 ± 0.0007	0.3083	0.0054 ± 0.0010	0.3761	nmol/μg	F (2, 27) = 1.882	NO
Serotonin (5-HT)	0.0086 ± 0.0022	0.0051 ± 0.0010	0.2629	0.0138 ± 0.0027	0.1009	ng/μg	F (2, 27) = 4.091	YES
Norepinephrine	0.0013 ± 0.0004	0.0007 ± 0.0002	0.2669	0.0033 ± 0.0004	0.0008 ***	ng/μg	F (2, 27) = 13.07	YES
ROS	0.1864 ± 0.0465	0.1204 ± 0.0196	0.2376	0.3410 ± 0.0440	0.0087 **	U/mL	F (2, 27) = 8.586	YES
ACh	0.5470 ± 0.1099	0.3712 ± 0.0536	0.1991	0.7664 ± 0.1086	0.1120	ug/μg	F (2, 27) = 4.398	YES
Cortisol	1.1900 ± 0.2340	0.8416 ± 0.1621	0.2683	1.5870 ± 0.2474	0.2082	pg/μg	F (2, 27) = 2.932	NO
Glutamate	0.0054 ± 0.0013	0.0035 ± 0.0006	0.2452	0.0074 ± 0.0014	0.2218	ug/μg	F (2, 27) = 2.974	NO
Glycine	0.0321 ± 0.0056	0.0193 ± 0.0037	0.0644	0.0349 ± 0.0046	0.6765	ug/μg	F (2, 27) = 3.139	NO
Histamine	0.0129 ± 0.0038	0.0104 ± 0.0025	0.5926	0.0178 ± 0.0034	0.2979	ng/μg	F (2, 27) = 1.33	NO
Catecholamine	0.6510 ± 0.1080	0.4566 ± 0.0871	0.2636	0.9893 ± 0.1557	0.0572	ng/μg	F (2, 27) = 5.012	YES
Melatonin	0.0235 ± 0.0065	0.0155 ± 0.0034	0.2450	0.0335 ± 0.0037	0.1490	pg/μg	F (2, 27) = 3.59	YES
Epinephrine	0.0044 ± 0.0013	0.0032 ± 0.0007	0.4703	0.0075 ± 0.0013	0.0693	ng/μg	F (2, 27) = 3.667	YES

**Table 3:** DNA methylation counts and differentially methylated regions (DMR) in zebrafish with different genetic backgrounds for either wild type (WT), *dnmt3aa* KO, or *dnmt3ab* KO zebrafish.

Categories	DNA Methylation Counts			Differentially Methylated Regions (DMRs)		
	WT	*dnmt3aa* KO	*dnmt3ab* KO	WT vs *dnmt3aa* KO	WT vs *dnmt3ab* KO	*dnmt3aa* KO *vs dnmt3ab* KO
Exonic	2,176,682	2,186,207	2,183,519	640	430	613
Intergenic	14,826,766	1,5002,735	14,869,769	6279	3740	6215
Intronic	17,219,672	17,423,241	17,313,624	6870	4035	6622
Splicing	6,211	6,276	6,225	4	4	4
Upstream	533,791	538,935	535,204	1038	651	963
Downstream	471,226	475,277	472,228	322	195	318
UTR3	568,462	572,270	569,330	287	161	273
UTR5	331,319	332,694	331,889	436	280	459
Total	36,166,511	36,570,202	36,314,198	15,962	9543	15,554

**Table 4:** Summary of *dnmt3aa* and *dnmt3ab* KO zebrafish phenotypes collected at morphological, biochemical, and behavioral levels compared to the control group. The signatures of the zebrafish behavioral and biochemical tests are summarized (↓: downregulated, ↑: upregulated).

Fish Lines	*dnmt3aa* KO Fish	*dnmt3ab* KO Fish
Morphological analysis		
Morphometric analysis	unaltered	unaltered
Biochemical analysis		
5-hmC/5-mC ratio	unaltered	unaltered
Neurotransmitters in the brain	unaltered	unaltered
Neurotransmitters in the whole body	↓	↑
Behavioral analysis		
Locomotor Activity in Novel Environment	unaltered	abnormal
Exploratory Behavior in Novel Environment	↓	unaltered
Aggressiveness	unaltered	↓
Predator Avoidance	↓	↓
Social Interaction	unaltered	↓
Shoaling	unaltered	loosen
Circadian Rhythm Locomotor Activity (Light Cycle)	unaltered	unaltered
Circadian Rhythm Locomotor Activity (Dark Cycle)	↑	↑
Color preference index ranking	unaltered	dysregulated
Short-term memory	↓	↓

## Supplementary Materials

The following are available online at https://www.mdpi.com/2073-4425/11/11/1322/s1, Supplementary data: Video S1. Comparison of the novel tank exploration behavior between wild type, *dnmt3aa*, and *dnmt3ab* KO fish. Video was played at 5× speed. Video S2. Comparison of the mirror biting behavior between wild type, *dnmt3aa,* and *dnmt3ab* KO fish. Video was played at 10× speed. Video S3. Comparison of the predator avoidance behavior between wild type, *dnmt3aa,* and *dnmt3ab* KO fish. Video was played at 10× speed. Video S4. Comparison of the conspecific interaction behavior between wild type, *dnmt3aa,* and *dnmt3ab* KO fish. Video was played at 10× speed. Video S5. Comparison of the shoaling behavior (three fish in a group) between wild type, *dnmt3aa,* and *dnmt3ab* KO fish. Video was played at 10× speed. Video S6. Comparison of the circadian rhythm locomotor activity (three fish in a group) behavior between wild type, *dnmt3aa*, and *dnmt3ab* KO fish. Video was played at 3× speed. Table S1. Top 30 genes that consisted of most DMRs between wild type and *dnmt3aa* KO zebrafish. Table S2. Top 30 genes that consisted of most DMRs between wild type and *dnmt3ab* KO zebrafish. Table S3. Top 30 genes that consisted of most DMRs between *dnmt3aa* KO and *dnmt3aa* KO zebrafish. Table S4. Annotated pathways for the top 30 DMR sites between groups using DAVID prediction.

## Appendix A

**Table A1:** The statistic details of each statistic test from each data (Column Factor = fish line factor, Row Factor = time interval factor, n.s. = not significant, * *p* < 0.05, ** *p* < 0.01, *** *p* < 0.001, **** *p* < 0.0001).

Novel Tank Test						
Behavior Endpoints	Fish Lines	Source of Variation	F (DFn, DFd)		*p* Value	Significance
Average Speed	*dnmt3aa* KO	Column Factor	F(1, 35) = 0.02003		0.8883	n.s.
		Row Factor	F(3.265, 114.3) = 2.265		0.0794	n.s.
	*dnmt3ab* KO	Column Factor	F(1, 58) = 0.0001992		0.9888	n.s.
		Row Factor	F(4.358, 252.7) = 10.57		<0.0001	****
Freezing Time Movement Ratio	*dnmt3aa* KO	Column Factor	F(1, 35) = 0.003813		0.9511	n.s.
		Row Factor	F(1.745, 61.06) = 0.1133		0.8672	n.s.
	*dnmt3ab* KO	Column Factor	F(1, 58) = 1.195		0.2788	n.s.
		Row Factor	F(3.281, 190.3) = 4.759		0.0024	**
Time in Top Duration	*dnmt3aa* KO	Column Factor	F(1, 35) = 18.70		0.0001	***
		Row Factor	F(3.828, 134) = 1.548		0.1941	n.s.
	*dnmt3ab* KO	Column Factor	F(1, 58) = 2.380		0.1284	n.s.
		Row Factor	F(4.707, 273) = 10.97		<0.0001	****
Number of Entries to the Top	*dnmt3aa* KO	Column Factor	F(1, 35) = 7.937		0.0079	**
		Row Factor	F(3.492, 122.2) = 2.944		0.0287	*
	*dnmt3ab* KO	Column Factor	F(1, 58) = 0.9411		0.3360	n.s.
		Row Factor	F(4.781, 277.3) = 6.991		<0.0001	****
Latency to Enter the Top	*dnmt3aa* KO	Column Factor	F(1, 35) = 36.68		<0.0001	****
		Row Factor	F(4.262, 149.2) = 7.211		<0.0001	****
	*dnmt3ab* KO	Column Factor	F(1, 58) = 2.980		0.0896	n.s.
		Row Factor	F(4.675, 271.1) = 21.92		<0.0001	****
Total Distance Traveled in the Top	*dnmt3aa* KO	Column Factor	F(1, 35) = 14.51		0.0005	***
		Row Factor	F(3.980, 139.3) = 1.620		0.1728	n.s.
	*dnmt3ab* KO	Column Factor	F(1, 58) = 2.387		0.1278	n.s.
		Row Factor	F(5.290, 306.8) = 8.278		<0.0001	****
Mirror Biting Test						
Behavior Endpoints		Fish Lines	U-Value		*p* Value	Significance
Mirror Biting Time Percentage		*dnmt3aa* KO	110		0.2212	n.s.
		*dnmt3ab* KO	161.5		< 0.0001	****
Longest Duration in the Mirror Side		*dnmt3aa* KO	144		0.8610	n.s.
		*dnmt3ab* KO	164.5		< 0.0001	****
Average Speed		*dnmt3aa* KO	87		0.0498	*
		*dnmt3ab* KO	384		0.3354	n.s.
Freezing Time Movement Ratio		*dnmt3aa* KO	117.5		0.3190	n.s.
		*dnmt3ab* KO	417.5		0.6359	n.s.
Swimming Time Movement Ratio		*dnmt3aa* KO	117.5		0.3183	n.s.
		*dnmt3ab* KO	416.5		0.6254	n.s.
Rapid Time Movement Ratio		*dnmt3aa* KO	85		0.0417	*
		*dnmt3ab* KO	369		0.2343	n.s.
Predator Avoidance Test						
Behavior Endpoints		Fish Lines	U-Value		*p* Value	Significance
Approaching Predator Time		*dnmt3aa* KO	53		0.0016	**
		*dnmt3ab* KO	42		< 0.0001	****
Average Distance to Separator		*dnmt3aa* KO	19		< 0.0001	****
		*dnmt3ab* KO	29		< 0.0001	****
Average Speed		*dnmt3aa* KO	119		0.3464	n.s.
		*dnmt3ab* KO	98		0.0218	*
Freezing Time Movement Ratio		*dnmt3aa* KO	71		0.0123	*
		*dnmt3ab* KO	78.50		0.0038	**
Swimming Time Movement Ratio		*dnmt3aa* KO	48		0.0009	***
		*dnmt3ab* KO	79.50		0.0042	**
Rapid Time Movement Ratio		*dnmt3aa* KO	124		0.4271	n.s.
		*dnmt3ab* KO	156.5		0.5224	n.s.
Social Interaction Test						
Behavior Endpoints		Fish Lines	U-Value		*p* Value	Significance
Average Speed		*dnmt3aa* KO	66		0.1379	n.s.
		*dnmt3ab* KO	78		0.0037	**
Interaction Time Percentage		*dnmt3aa* KO	95		0.7186	n.s.
		*dnmt3ab* KO	146		0.3559	n.s.
Longest Duration in Separator Side		*dnmt3aa* KO	80		0.3500	n.s.
		*dnmt3ab* KO	174		0.8800	n.s.
Average Distance to Separator		*dnmt3aa* KO	73		0.2274	n.s.
		*dnmt3ab* KO	79		0.0041	**
Shoaling Test						
Behavior Endpoints		Fish Lines	U-Value		*p* Value	Significance
Average Inter-Fish Distance		*dnmt3aa* KO	88		0.1226	n.s.
		*dnmt3ab* KO	60		0.0005	***
Average Shoal Area		*dnmt3aa* KO	8		0.2867	n.s.
		*dnmt3ab* KO	0		0.0020	**
Average Nearest Neighbor Distance		*dnmt3aa* KO	80		0.0687	n.s.
		*dnmt3ab* KO	43		< 0.0001	****
Average Farthest Neighbor Distance		*dnmt3aa* KO	91		0.1494	n.s.
		*dnmt3ab* KO	29		< 0.0001	****
Average Speed		*dnmt3aa* KO	79		0.0636	n.s.
		*dnmt3ab* KO	81		0.0050	**
Time in Top Duration		*dnmt3aa* KO	12		< 0.0001	****
		*dnmt3ab* KO	158		0.5544	n.s.
Average Distance to Center of the Tank		*dnmt3aa* KO	60		0.0111	*
		*dnmt3ab* KO	95		0.0171	*
Circadian Rhythm Locomotor Activity Test						
Cycles	Behavior Endpoints	Fish Lines	U-Value		*p* Value	Significance
Day	Average Speed	*dnmt3aa* KO	49,862		0.0620	n.s.
		*dnmt3ab* KO	35,242		0.5673	n.s.
	Average Angular Velocity	*dnmt3aa* KO	31,207		0.0055	**
		*dnmt3ab* KO	13,886		0.0002	***
	Meandering	*dnmt3aa* KO	17,567		0.6189	n.s.
		*dnmt3ab* KO	32,656		0.0470	*
Night	Average Speed	*dnmt3aa* KO	16,195		< 0.0001	****
		*dnmt3ab* KO	4077		< 0.0001	****
	Average Angular Velocity	*dnmt3aa* KO	31,267		< 0.0001	****
		*dnmt3ab* KO	27,105		< 0.0001	****
	Meandering	*dnmt3aa* KO	26,506		< 0.0001	****
		*dnmt3ab* KO	11,843		< 0.0001	****
Color Preference Test						
Color Combinations	Fish Line Comparisons		F (DFn, DFd)	Colors	*p* Value	Significance
Green-Blue	WT vs. *dnmt3aa* KO		F(5, 124) = 22.88	Green	0.0016	**
	WT vs. *dnmt3ab* KO				< 0.0001	****
	*dnmt3aa* KO vs. *dnmt3ab* KO				> 0.9999	n.s.
	WT vs. *dnmt3aa* KO			Blue	0.0024	**
	WT vs. *dnmt3ab* KO				< 0.0001	****
	*dnmt3aa* KO vs. *dnmt3ab* KO				0.2146	n.s.
Green-Yellow	WT vs. *dnmt3aa* KO		F(5, 124) = 182.4	Green	0.0016	**
	WT vs. *dnmt3ab* KO				< 0.0001	****
	*dnmt3aa* KO vs. *dnmt3ab* KO				< 0.0001	****
	WT vs. *dnmt3aa* KO			Yellow	0.0016	**
	WT vs. *dnmt3ab* KO				< 0.0001	****
	*dnmt3aa* KO vs. *dnmt3ab* KO				< 0.0001	****
Red-Blue	WT vs. *dnmt3aa* KO		F(5, 124) = 35.28	Red	> 0.9999	n.s.
	WT vs. *dnmt3ab* KO				0.9273	n.s.
	*dnmt3aa* KO vs. *dnmt3ab* KO				0.9658	n.s.
	WT vs. *dnmt3aa* KO			Blue	> 0.9999	n.s.
	WT vs. *dnmt3ab* KO				0.9274	n.s.
	*dnmt3aa* KO vs. *dnmt3ab* KO				0.9658	n.s.
Green-Red	WT vs. *dnmt3aa* KO		F(5, 124) = 62.59	Green	0.9984	n.s.
	WT vs. *dnmt3ab* KO				< 0.0001	****
	*dnmt3aa* KO vs. *dnmt3ab* KO				< 0.0001	****
	WT vs. *dnmt3aa* KO			Red	0.9984	n.s.
	WT vs. *dnmt3ab* KO				< 0.0001	****
	*dnmt3aa* KO vs. *dnmt3ab* KO				< 0.0001	****
Red-Yellow	WT vs. *dnmt3aa* KO		F(5, 124) = 381.1	Red	0.0015	**
	WT vs. *dnmt3ab* KO				< 0.0001	****
	*dnmt3aa* KO vs. *dnmt3ab* KO				0.8337	n.s.
	WT vs. *dnmt3aa* KO			Yellow	0.0015	**
	WT vs. *dnmt3ab* KO				< 0.0001	****
	*dnmt3aa* KO vs. *dnmt3ab* KO				0.8337	n.s.
Blue-Yellow	WT vs. *dnmt3aa* KO		F(5, 124) = 934.6	Blue	< 0.0001	****
	WT vs. *dnmt3ab* KO				< 0.0001	****
	*dnmt3aa* KO vs. *dnmt3ab* KO				0.3531	n.s.
	WT vs. *dnmt3aa* KO			Yellow	< 0.0001	****
	WT vs. *dnmt3ab* KO				< 0.0001	****
	*dnmt3aa* KO vs. *dnmt3ab* KO				0.3531	n.s.
Short-Term Memory Test						
Behavior Endpoints		Fish Line Comparisons	F (DFn, DFd)		*p* Value	Significance
Latency After 24 h	Before Training	WT vs. *dnmt3aa* KO	Column Factor F (2, 86) = 3.487 *p* = 0.0350 * Row Factor F (1, 86) = 24.59 *p* < 0.0001 ****		0.9993	n.s.
		WT vs. *dnmt3ab* KO			> 0.9999	n.s.
		*dnmt3aa* KO vs. *dnmt3ab* KO			0.9994	n.s.
	After Training	WT vs. *dnmt3aa* KO			0.0011	**
		WT vs. *dnmt3ab* KO			0.0443	*
		*dnmt3aa* KO vs. *dnmt3ab* KO			0.4340	n.s.
Global DNA Methylation Level Test						
Parameters	Fish Line Comparisons		F (DFn, DFd)		*p* Value	Significance
5-hmC	WT vs. *dnmt3aa* KO		F (2, 12) = 0.9873		0.2012	n.s.
	WT vs. *dnmt3ab* KO				0.3745	n.s.
	*dnmt3aa* KO vs. *dnmt3ab* KO				0.6478	n.s.
5-mC	WT vs. *dnmt3aa* KO		F (2, 12) = 1.439		0.1761	n.s.
	WT vs. *dnmt3ab* KO				0.1788	n.s.
	*dnmt3aa* KO vs. *dnmt3ab* KO				0.9263	n.s.
Ratio	WT vs. *dnmt3aa* KO		F (2, 12) = 0.006109		0.9247	n.s.
	WT vs. *dnmt3ab* KO				0.9299	n.s.
	*dnmt3aa* KO vs. *dnmt3ab* KO				0.9908	n.s.
