# Supplementary material for: Duplicated dnmt3aa and dnmt3ab DNA Methyltransferase Genes Play Essential and Non-Overlapped Functions on Modulating Behavioral Control in Zebrafish
Source: Genes (Basel). 2020 Nov 7;11(11):1322. doi: 10.3390/genes11111322 (PMC7695179; doi:10.3390/genes11111322)
Supplement: Supplementary file 1 [file genes-11-01322-s001.zip › [dnmt3aa and dnmt3ab videos]/Table S1 to S4.docx]

**Table S1.** Top thirty genes that consisted most DMRs between wild type and *dnmt3aa* KO zebrafish

| ranking | chromosome | DMRs | delta beta | P value | feature | Gene symbol |
| --- | --- | --- | --- | --- | --- | --- |
| 1 | 25 | 367 | -0.1643129 | 2.00E-111 | intronic | *isl2a* |
| 2 | 24 | 259 | -0.1913038 | 1.19E-84 | intergenic | *pip4k2aa* |
| 3 | 7 | 234 | -0.1185483 | 2.25E-43 | exonic | *si:ch1073-159d7.5* |
| 4 | 7 | 231 | -0.1775301 | 9.33E-82 | exonic | *si:ch1073-159d7.13* |
| 5 | 25 | 212 | -0.1670926 | 1.39E-53 | downstream | *SCAPER* |
| 6 | 22 | 210 | -0.1453841 | 1.26E-52 | intergenic | *TMEM161A* |
| 7 | 7 | 203 | -0.1360453 | 4.46E-33 | intronic | *irx6a* |
| 8 | 7 | 199 | -0.1173595 | 4.43E-37 | upstream | *pax6b* |
| 9 | 7 | 197 | -0.1466783 | 9.29E-69 | intergenic | *hsd17b12b* |
| 10 | 2 | 196 | -0.1201347 | 1.65E-30 | exonic | *neurod6b* |
| 11 | 2 | 183 | -0.1638367 | 4.39E-38 | exonic | *HES6* |
| 12 | 14 | 176 | -0.103556 | 1.05E-35 | intergenic | *zgc:110843* |
| 13 | 17 | 176 | -0.1086103 | 1.47E-28 | downstream | *FOXA1* |
| 14 | 7 | 176 | -0.1407529 | 4.51E-31 | upstream | *irx5a* |
| 15 | 6 | 174 | -0.1489296 | 9.79E-40 | exonic | *dmbx1b* |
| 16 | 5 | 172 | -0.1844732 | 8.36E-65 | intergenic | *FOXD1* |
| 17 | 1 | 169 | -0.1206607 | 3.75E-21 | UTR5 | *MAB21L2* |
| 18 | 17 | 166 | -0.1231516 | 6.35E-28 | intergenic | *EHBP1* |
| 19 | 21 | 163 | -0.1450651 | 2.20E-32 | exonic | *gadd45gb.1* |
| 20 | 24 | 160 | -0.1958383 | 2.70E-50 | exonic | *PRDM14* |
| 21 | 1 | 156 | -0.1340295 | 4.20E-25 | UTR3 | *uncx4.1* |
| 22 | 9 | 155 | -0.115141 | 5.58E-31 | intronic | *NHLH2* |
| 23 | 16 | 150 | -0.1193718 | 9.87E-17 | UTR3 | *irx2a* |
| 24 | 4 | 149 | -0.1262627 | 1.87E-35 | intergenic | *NET1* |
| 25 | 2 | 145 | -0.1027376 | 4.13E-27 | intergenic | *PTF1A* |
| 26 | 22 | 138 | -0.1693915 | 1.87E-25 | intronic | *BCOR* |
| 27 | 1 | 137 | -0.1301141 | 1.01E-26 | upstream | *TENM3* |
| 28 | 15 | 137 | -0.1611362 | 1.12E-23 | intergenic | *auts2b* |
| 29 | 23 | 136 | -0.1315105 | 1.24E-29 | upstream | *mafba* |
| 30 | 23 | 136 | -0.17271 | 6.16E-30 | intergenic | *BHLHE23* |

**Table S2.** Top thirty genes that consisted most DMRs between wild type and *dnmt3ab* KO zebrafish

| ranking | chromosome | DMRs | delta beta | P value | feature | Gene symbol |
| --- | --- | --- | --- | --- | --- | --- |
| 1 | 7 | 196 | -0.1263926 | 6.03E-44 | exonic | *march3* |
| 2 | 7 | 196 | -0.1766668 | 3.87E-65 | exonic | *sept15* |
| 3 | 8 | 189 | 0.14067047 | 2.94E-28 | intergenic | *AACS* |
| 4 | 5 | 178 | -0.1584721 | 5.02E-46 | intergenic | *AADAT* |
| 5 | 25 | 143 | -0.187782 | 1.68E-57 | intronic | *aak1a* |
| 6 | 6 | 142 | 0.12709248 | 9.92E-17 | intronic | *AAMP* |
| 7 | 14 | 137 | -0.1182284 | 1.36E-22 | exonic | *AANAT1* |
| 8 | 22 | 135 | 0.12349375 | 3.13E-19 | intronic | *abca2* |
| 9 | 14 | 132 | -0.1907897 | 3.35E-41 | intergenic | *ABCB5* |
| 10 | 14 | 131 | -0.1007901 | 2.26E-18 | intergenic | *ABCB5* |
| 11 | 10 | 124 | -0.1491373 | 6.82E-47 | intronic | *ABCB5* |
| 12 | 20 | 115 | 0.18455056 | 3.10E-43 | intronic | *ABCB5* |
| 13 | 23 | 115 | -0.1093954 | 3.39E-14 | exonic | *ABCC3* |
| 14 | 17 | 114 | -0.1164132 | 1.73E-14 | exonic | *ablim1a* |
| 15 | 9 | 114 | -0.1970942 | 8.76E-47 | intronic | *ACBD6* |
| 16 | 16 | 108 | -0.1030858 | 1.59E-12 | UTR5 | *aclya* |
| 17 | 5 | 108 | -0.1599554 | 1.07E-27 | intronic | *acsl2* |
| 18 | 17 | 105 | -0.1711476 | 3.37E-28 | intronic | *ACSS2* |
| 19 | 13 | 102 | -0.1565244 | 1.33E-32 | intergenic | *acvr1bb* |
| 20 | 5 | 101 | -0.134664 | 2.70E-20 | UTR5 | *ACY1* |
| 21 | 13 | 98 | 0.12701665 | 6.01E-38 | exonic | *ADAM12* |
| 22 | 19 | 98 | -0.1787016 | 4.13E-15 | UTR5 | *adam17b* |
| 23 | 18 | 97 | 0.17902483 | 2.88E-25 | intergenic | *ADAM22* |
| 24 | 7 | 96 | -0.1072784 | 1.49E-32 | intronic | *ADAP2* |
| 25 | 24 | 94 | 0.17103099 | 3.68E-15 | intergenic | *ADAT1* |
| 26 | 23 | 94 | 0.15325691 | 2.44E-25 | upstream | *adck5* |
| 27 | 7 | 93 | -0.1026049 | 4.93E-31 | intronic | *add3a* |
| 28 | 9 | 93 | -0.1248006 | 4.08E-14 | UTR3 | *adgrb1a* |
| 29 | 5 | 92 | -0.1274696 | 4.20E-15 | intergenic | *adgrb1b* |
| 30 | 21 | 92 | -0.1923553 | 2.11E-27 | upstream | *ADGRG3* |

**Table S3.** Top thirty genes that consisted most DMRs between *dnmt3aa* KO and *dnmt3aa* KO zebrafish.

| ranking | chromosome | DMRs | delta beta | P value | feature | Gene symbol |
| --- | --- | --- | --- | --- | --- | --- |
| 1 | 17 | 448 | 0.12327324 | 4.05E-77 | UTR3 | *OTX2* |
| 2 | 24 | 267 | 0.17655579 | 4.29E-67 | intergenic | *pip4k2aa* |
| 3 | 3 | 261 | 0.1256534 | 3.47E-49 | UTR5 | *UNCX* |
| 4 | 21 | 234 | 0.17520138 | 3.90E-91 | intergenic | *TBCA* |
| 5 | 17 | 226 | 0.1856763 | 2.83E-99 | exonic | *pax1a* |
| 6 | 7 | 224 | 0.15306959 | 1.65E-45 | intronic | *irx6a* |
| 7 | 24 | 214 | 0.19781049 | 2.47E-72 | exonic | *PRDM14* |
| 8 | 24 | 204 | 0.12474178 | 3.45E-44 | exonic | *neurod6a* |
| 9 | 7 | 182 | 0.171557 | 4.39E-44 | upstream | *irx5a* |
| 10 | 22 | 180 | 0.17520597 | 1.21E-47 | intronic | *LHX9* |
| 11 | 25 | 179 | 0.15719181 | 1.35E-49 | intronic | *isl2a* |
| 12 | 24 | 168 | 0.17482809 | 5.32E-65 | intergenic | *MLLT10* |
| 13 | 15 | 168 | 0.16924318 | 2.02E-39 | intergenic | *lhx1a* |
| 14 | 2 | 168 | 0.16356369 | 6.81E-35 | exonic | *HES6* |
| 15 | 8 | 163 | 0.15835385 | 8.56E-26 | intergenic | *DALRD3* |
| 16 | 19 | 163 | 0.1055308 | 1.32E-15 | UTR5 | *scrt1a* |
| 17 | 15 | 159 | 0.14036506 | 7.14E-31 | intronic | *lhx1a* |
| 18 | 5 | 157 | 0.11477867 | 7.60E-31 | intronic | *tet3* |
| 19 | 20 | 154 | 0.15867702 | 1.73E-44 | exonic | *foxc1b* |
| 20 | 19 | 152 | 0.12958623 | 1.05E-38 | intronic | *TFAP2E* |
| 21 | 23 | 150 | 0.1339986 | 1.67E-34 | upstream | *mafba* |
| 22 | 25 | 147 | 0.11285738 | 5.18E-18 | upstream | *DEAF1* |
| 23 | 3 | 146 | 0.10382803 | 1.06E-20 | intronic | *hoxb3a* |
| 24 | 14 | 145 | 0.17098439 | 7.52E-38 | intergenic | *nkx3.2* |
| 25 | 17 | 142 | 0.10598007 | 5.41E-19 | intergenic | *EHBP1* |
| 26 | 6 | 141 | 0.14712124 | 6.09E-28 | intronic | *zeb2b* |
| 27 | 23 | 138 | 0.14582363 | 1.88E-33 | intronic | *myt1b* |
| 28 | 19 | 138 | 0.14449179 | 7.59E-27 | intergenic | *twist1a* |
| 29 | 13 | 136 | 0.13331421 | 1.15E-30 | intronic | *PITX3* |
| 30 | 7 | 135 | 0.19503815 | 2.90E-34 | intergenic | *irx6a* |

**Table S4.** Annotated pathways for top thirty DMR sites between groups using DAVID prediction

| Annotation | Gene | P value | Fold enrichment | FDR |
| --- | --- | --- | --- | --- |
| WT vs *dnmt3aa* KO | | | | |
| Regulation of transcription, DNA templated | *bcor, isl2a, uncx4.1, bhlhe23, dmbx1b, foxa1, foxd1, hes6, irx2a, irx5a, irx6a, neuro6b, pax6b, ptf1a, mafba* | 2.5E-10 | 7.4 | 2.6E-7 |
| Multicellular organism development | *isl2a, uncx4.1, dmbx1b, mab21l2, neurod6b, pax6b* | 1.1E-3 | 6.9 | 1.2E0 |
| Cell differentiation | *uncx4.1, neurod6b, ptf1a, tenm3* | 4.5E-3 | 11.3 | 4.6E0 |
| Lens development | *mab21l2, pax6b* | 3.5E-2 | 54.0 | 3.1E1 |
| Retina morphogenesis | *bcor, irx2a* | 4.9E-2 | 38.1 | 4.1E1 |
| Exocrine pancreas development | *isl2a, ptf1a* | 5.6E-2 | 33.2 | 4.5E1 |
| Hindbrain development | *pax6b, ptf1a* | 6.3E-2 | 29.5 | 4.9E1 |
| Anterior/posterior pattern specification | *pax6b, mafba* | 7.1E-2 | 25.9 | 5.4E1 |
| Regulation of cell cycle | *dmbx1b, gadd45gb.1* | 9.0E-2 | 20.3 | 6.3E1 |
| WT vs *dnmt3ab* KO | | | | |
| Regulation of transcription, DNA templated | *isl2a, lmx1ba, tsc22d1, bhlhe22, foxd1, fosl2* | 2.8E-3 | 3.8 | 2.8E0 |
| *dnmt3aa* KO vs *dnmt3ab* KO | | | | |
| Regulation of transcription, DNA templated | *isl2a, lhx1a, lhx9, nkx3.2, uncx, foxc1b, hes6, hoxb3a, irx5a, irx6a, myt1b, neurod6b, otx2, pax1a, pitx3, tfap2e, mafba* | 1.1E-11 | 7.4 | 1.2E-8 |
| Multicellular organism development | *isl2a, lhx1a, hoxb3a, neurod6b, otx2, pax1a, pitx3* | 2.6E-4 | 7.2 | 2.9E-1 |
| Negative regulation of transcription, DNA templated | *ihx1a, ihx9, hes6* | 1.1E-2 | 18.1 | 1.2E1 |
| Neuron fate specification | *isl2a, lhx9* | 1.7E-2 | 114.7 | 1.7E1 |
| Cerebellum development | *lhx1a, otx2* | 3.6E-2 | 52.1 | 3.4E1 |
| Embryonic pattern specification | *lhx1a, mafba* | 4.1E-2 | 45.9 | 3.7E1 |
| motor neuron axon guidance | *lhx1a, lhx9* | 5.6E-2 | 33.7 | 4.7E1 |
| camera-type eye development | *foxc1b, pitx3* | 8.1E-2 | 22.9 | 6.1E1 |
| Anterior/posterior pattern specification | *lhx1a, mafba* | 8.1E-2 | 22.9 | 6.1E-1 |
